# Chromatin changes in *Anopheles gambiae* induced by *Plasmodium falciparum* infection

**DOI:** 10.1186/s13072-018-0250-9

**Published:** 2019-01-07

**Authors:** José L. Ruiz, Rakiswendé S. Yerbanga, Thierry Lefèvre, Jean B. Ouedraogo, Victor G. Corces, Elena Gómez-Díaz

**Affiliations:** 1 Estación Biológica de Doñana (EBD), Consejo Superior de Investigaciones Científicas, 41092 Seville, Spain; 2 Instituto de Parasitología y Biomedicina López-Neyra (IPBLN), Consejo Superior de Investigaciones Científicas, 18016 Granada, Spain; 3 Institut de Recherche en Sciences de la Santé (IRSS), 01 BP 171, Bobo Dioulasso, Burkina Faso; 4 MIVEGEC, IRD, CNRS, University of Montpellier, Montpellier, France; 5 Department of Biology, Emory University, 1510 Clifton Road NE, Atlanta, GA 30322 USA

**Keywords:** Human malaria, Mosquitoes, Histone modifications, Gene regulation, ChIP-seq, RNA-seq, Transcriptome, Epigenome

## Abstract

**Background:**

Infection by the human malaria parasite leads to important changes in mosquito phenotypic traits related to vector competence. However, we still lack a clear understanding of the underlying mechanisms and, in particular, of the epigenetic basis for these changes. We have examined genome-wide distribution maps of H3K27ac, H3K9ac, H3K9me3 and H3K4me3 by ChIP-seq and the transcriptome by RNA-seq, of midguts from *Anopheles gambiae* mosquitoes blood-fed uninfected and infected with natural isolates of the human malaria parasite *Plasmodium falciparum* in Burkina Faso.

**Results:**

We report 15,916 regions containing differential histone modification enrichment between infected and uninfected, of which 8339 locate at promoters and/or intersect with genes. The functional annotation of these regions allowed us to identify infection-responsive genes showing differential enrichment in various histone modifications, such as CLIP proteases, antimicrobial peptides-encoding genes, and genes related to melanization responses and the complement system. Further, the motif analysis of regions differentially enriched in various histone modifications predicts binding sites that might be involved in the cis-regulation of these regions, such as Deaf1, Pangolin and Dorsal transcription factors (TFs). Some of these TFs are known to regulate immunity gene expression in *Drosophila* and are involved in the Notch and JAK/STAT signaling pathways.

**Conclusions:**

The analysis of malaria infection-induced chromatin changes in mosquitoes is important not only to identify regulatory elements and genes underlying mosquito responses to *P. falciparum* infection, but also for possible applications to the genetic manipulation of mosquitoes and to other mosquito-borne systems.

**Electronic supplementary material:**

The online version of this article (10.1186/s13072-018-0250-9) contains supplementary material, which is available to authorized users.

## Background

Mosquitoes are the most medically important arthropod vectors of disease. Among mosquito-borne diseases, malaria, caused by protozoan parasites of the genus *Plasmodium*, is the deadliest. The human malaria parasite *P. falciparum* is the most prevalent agent in Africa, and it is responsible for at least 200 million acute cases worldwide and around half a million deaths each year [1]. It is transmitted by *Anopheles* mosquitoes, *A. gambiae* being the main vector. At the molecular level, *P. falciparum* infection induces drastic and rapid changes in gene expression in mosquito tissues that relate to functions involved in immunity, development, physiology and reproduction [2]. The immune response during infection is the best characterized of these pathways and mostly occurs in the midgut of infected mosquitoes. This involves, among others, the activation of genes related to epithelial nitration responses, melanization and the complement-like system [3–5]. Furthermore, infection in mosquitoes impacts epidemiologically important life-history traits such as vector competence, i.e., the ability of the mosquito to acquire, maintain and transmit the parasite, and to prime subsequent infections [6–8]. There is substantial variability in the responses of the mosquito to *P. falciparum* infection that depends on both genetic and environmental contexts [9, 10]. However, the mechanisms that regulate phenotypic responses to infection in mosquitoes, and that might mediate memory of the malaria infection phenotype, are little understood.

Chromatin-associated processes participate in the regulation of gene expression during development as well as in the differentiated tissues of the adult organism. These processes are sensitive to environmental stimuli and they are more or less transitory and can potentially be inherited, allowing living organisms and individual cells to continuously integrate internal and external inputs and to mediate responses through gene regulation [11, 12]. Among these, post-translational modifications of histones (hPTMs) impact the structure and/or function of chromatin, with different histone marks yielding distinct functional consequences [13]. For example, in *Drosophila*, as in other organisms, H3K27ac, H3K9ac and H3K4me3 are linked to gene activation and localize at promoters, whereas H3K9me3 is associated with silencing and occupies broader regions [14]. Various combinations of active and repressive histone modifications define chromatin states that are linked to gene function [15–17]. These modifications can remain during cell division, leaving a record of gene activity, i.e., epigenetic memory, that affects or primes the transcriptional response later in life [12, 18–24]. In mosquitoes, despite their relevance to human health, there is very little knowledge of chromatin regulation and its link to mosquito immunity, physiology and behavior [25]. In a previous study, we characterized genome-wide occupancy patterns of various histone modifications and established a link between hPTMs and gene expression profiles in midguts and salivary glands of the human malaria vector *A. gambiae* [26]. In addition, recent reports revealed the role of various transcription factors, such as REL2, lola and Deaf1, in *A. gambiae* immune defenses [27, 28]. These findings have set the stage for additional studies aimed at understanding how the chromatin landscape is altered in *P. falciparum*-infected mosquito tissues and what are the molecular players involved in these malaria-induced responses.

Available data on the phenotypic and transcriptional responses of mosquitoes to infection by *Plasmodium* are built on the use of mosquito–parasite combinations that in most cases differ from those found in nature. In these studies, infections often take place under standard laboratory conditions, using laboratory-adapted parasite clones and commercially available laboratory mosquito strains. Such experiments are useful to distinguish the contribution of parasite and mosquito genetic factors and also the influence of various environmental variables on the infection output, but they may not reflect the complexity of interactions that take place in natural conditions [29]. In this context, field-based studies are critical as they offer the advantage of allowing a more realistic picture of the molecular interactions in the context of natural transmission.

In this study, we aim to provide a comprehensive understanding of the chromatin and the transcriptional responses induced by *P. falciparum* infection of *A. gambiae* in the conditions found in a malaria endemic area in Africa. For this purpose, we compared genome-wide maps of histone modification profiles in infected and control mosquito tissues to identify chromatin state transitions associated with infection and examined the expression pattern of genes that annotate to regions containing differential histone modifications using the natural association between the mosquito *A. gambiae* and natural field isolates of the human malaria parasite *P. falciparum* in Burkina Faso. Motif enrichment analysis at these regulatory regions allowed us to predict the binding sites of several transcription factors, some of which have been shown to be involved in mosquito immune responses by previous studies.

## Results

### Chromatin states in malaria-infected mosquitoes

*Anopheles gambiae* were fed with blood obtained from malaria-infected human volunteers in Burkina Faso. Midguts from infected and uninfected (blood-fed uninfected) female mosquitoes were dissected and pooled separately for each condition (Additional file 1: Table S1). On the pooled samples, we carried out RNA-seq and ChIP-seq using antibodies to several histone modifications: H3K9ac, H3K27ac, H3K4me3 and H3K9me3 (Additional file 2: Table S2). Regions enriched in these histone modifications identified by MACS2 [30] are listed in Additional file 3: Table S3. We found similar numbers of peaks in the infected and uninfected mosquitoes and a high correlation in the ChIP-seq profiles (Additional file 4: Figure S1A), indicating that histone modification profiles are comparable between the two conditions. Based on this result, we focus particularly on characterizing functional chromatin states in the infected condition, and the results for the uninfected are given in supplemental material (Additional file 4: Figures S1, S2).

**Fig. 1:**
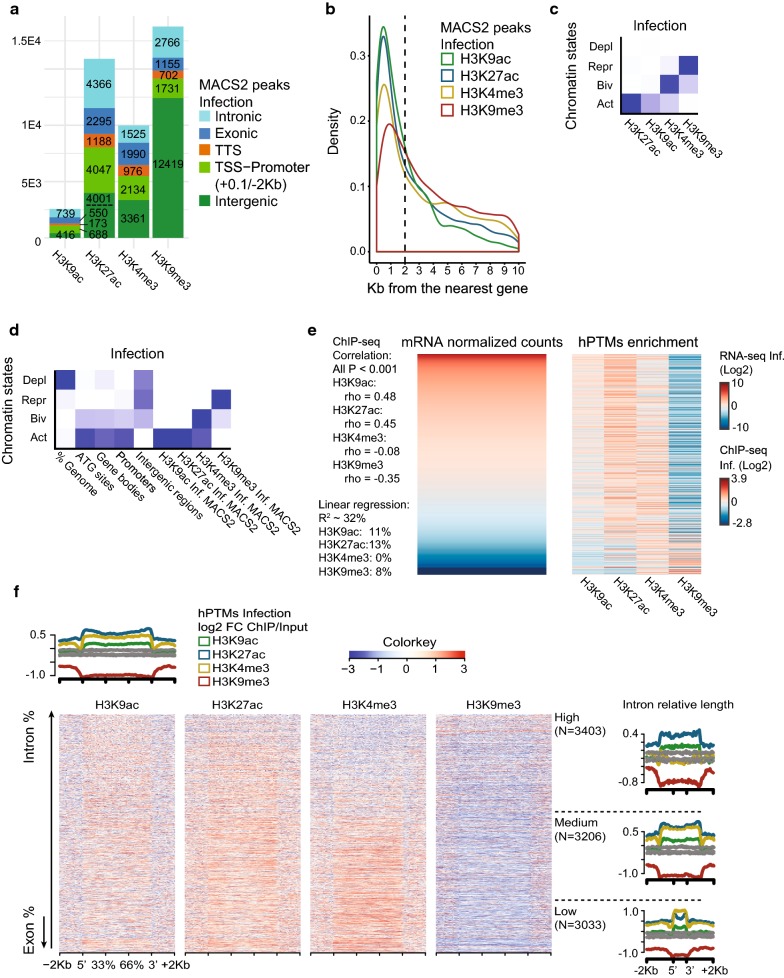
Association between histone modification profiles and gene expression regulation in *A. gambiae.* **a** Annotation of MACS2 ChIP-seq peaks for each histone modification to genomic features: TSS-Promoters, TTSs, Intergenic, Intron and Exon regions. The plot corresponds to the infected condition; data for the uninfected are given in Additional file 4: Figure S1. **b** Density plot showing the position (Kb upstream) of MACS2 peaks for each histone modification with respect to the ATG protein initiation codon of the nearest downstream gene. Same as above, data are for the infected condition. **c** Heatmap of emission parameters from ChromHMM analysis using a four chromatin states model based on histone modification enrichment patterns in the infected condition. The predicted states are: Deplet (depleted, low levels of all hPTMs), Repr (repressive, H3K9me3 enrichment), Biv (bivalent, H3K4me3/H3K9me3 enrichment) and Act (active, H3K27ac/H3K9ac/H3K4me3 enrichment). Darker blue indicates higher enrichment of a particular histone modification. **d** Heatmap showing the overlap of various genomic features, including MACS2 peaks located in promoters (2 Kb from the ATG) or gene bodies in the infected condition, with the predicted chromatin states. Darker blue in the first column indicates higher percentage of the genome overlapped by a given state. For other columns, it indicates the likelihood of finding a particular chromatin state in each genomic feature compared to what it would be expected by chance. **e** Heatmaps showing mRNA levels (left) and histone modification enrichment profiles (right) of genes displaying a MACS2 peak in the promoter or the gene body. Data correspond to the infected condition. Genes are ordered by mRNA levels. ChIP-seq enrichment at the promoters and the gene bodies is normalized (RPKM) and input-corrected. Data are log2-scaled and mean-centered. Spearman rank correlation coefficient (rho) and corresponding *P* value are shown for the association between each histone modification enrichment levels and mRNA levels. The variance in mRNA levels explained by the combined and individual enrichment levels of various histone modifications is shown, according to a linear regression model considering gene expression as response and ChIP-seq enrichment as covariate. **f** Heatmaps showing histone modification enrichment profiles at high and medium expressed genes in the infected condition. Genes are ordered by the percentage of the body containing introns and exons. Average profile plots show density of normalized (RPKM) and input-corrected ChIP-seq reads for each histone modification at high and medium expressed genes (top) and at those genes classified by the percentage of the gene body containing introns (right)

ChIP-seq peaks annotated to genomic features are shown in Fig. 1a and Additional file 4: Figure S1B. Results show that most ChIP-seq peaks correspond to H3K9me3-marked regions (37,343), followed by H3K27ac (35,217), H3K4me3 (19,945) and H3K9ac (6131) (Additional file 3: Table S3). The analysis of ChIP-seq peaks with respect to genomic features shows that upstream regions significantly enriched in histone modifications are mostly located in a 2 Kb window from the translation start codon (ATG) of *A. gambiae* genes, with lower enrichment at distances greater than 2 Kb. For example, in the infected samples, we find that 26,210 out of 47,228 total ChIP-seq peaks (55.5%) are located in the − 2 Kb gene body regions, whereas 1934 (4.10%) are located up to 2 Kb downstream from the gene bodies (Fig. 1b). A similar distribution and percentage of peaks for each distance class is reported in the uninfected mosquitoes (Additional file 4: Figure S1C). Based on this observation, we annotated peaks to the promoters of nearby genes when located less than 2 Kb upstream, and used this annotation for all subsequent analysis.

We used ChromHMM [31] to segment the genome into four distinct chromatin states based on relative enrichment levels of H3K9ac, H3K27ac, H3K4me3 and H3K9me3, that we named as follows: hPTM depleted, i.e., low levels of enrichment for all hPTMs, active (H3K9ac/H3K27ac and H3K4me3), repressed (H3K9me3) and bivalent (H3K4me3/H3K9me3) (Fig. 1c, Additional file 4: Figure S1D). As expected, most of the genome is in a depleted state, whereas gene bodies and promoters display enrichment in active state (Fig. 1d, Additional file 4: Figure S1E). Following this categorization, we investigated the association between various combinations of active and repressive histone modifications at promoters and gene bodies, and gene expression by RNA-seq. As expected, active chromatin is associated with expressed genes, whereas chromatin marked with H3K9me3 associates with silent or low-expressed genes. Genes showing a bivalent enrichment pattern (H3K4me3 and H3K9me3) are generally expressed at low levels (Fig. 1e, Additional file 4: Figure S2A). This is corroborated by a positive and statistically significant correlation between levels of enrichment in active histone modifications (H3K9ac, H3K27ac) and mRNA levels of ChIP-seq peaks annotated genes, whereas the correlation is negative for H3K9me3 (Fig. 1e, Additional file 4: Figure S2A). When analyzed together in a linear regression analysis, the combined histone modifications explained ~ 32% of the variance in mRNA levels in the infected condition and ~ 27% in the uninfected. This analysis also suggests that H3K9ac and H3K27ac are better predictors of mRNA levels, compared to H3K4me3 and H3K9me3 (Fig. 1e, Additional file 4: Figure S2A). Following what has been described for *Drosophila* [32], we classified genes according to the gene structure, i.e., length of the coding region and length of intronic segments. We observed that expressed genes with long introns and relatively short coding regions show broad H3K27ac and H3K9ac domains. In contrast, expressed genes with more uniformly distributed coding regions show a more localized H3K27ac/H3K9ac enrichment through the gene body and higher enrichment in H3K4me3 (Fig. 1f, Additional file 4: Figure S2B).

In addition to peaks that annotate to gene bodies and promoters, we report more distal regions (> 2 Kb from the translation start codon of the nearest downstream gene) significantly enriched in H3K27ac (MACS2 peaks) but depleted in H3K9ac/H3K4me3 that are the hallmark histone modifications for active promoters (3081 and 3586 peaks in infected and control conditions, respectively) (Additional file 3: Table S3). We also observe in the set of distal H3K27ac peaks the presence of RNA-seq reads mapped, indicating that they could correspond to enhancer-like regulatory sites (Additional file 4: Figure S3).

### Regions differentially enriched in histone modifications associated with *P. falciparum* infection

**Fig. 2:**
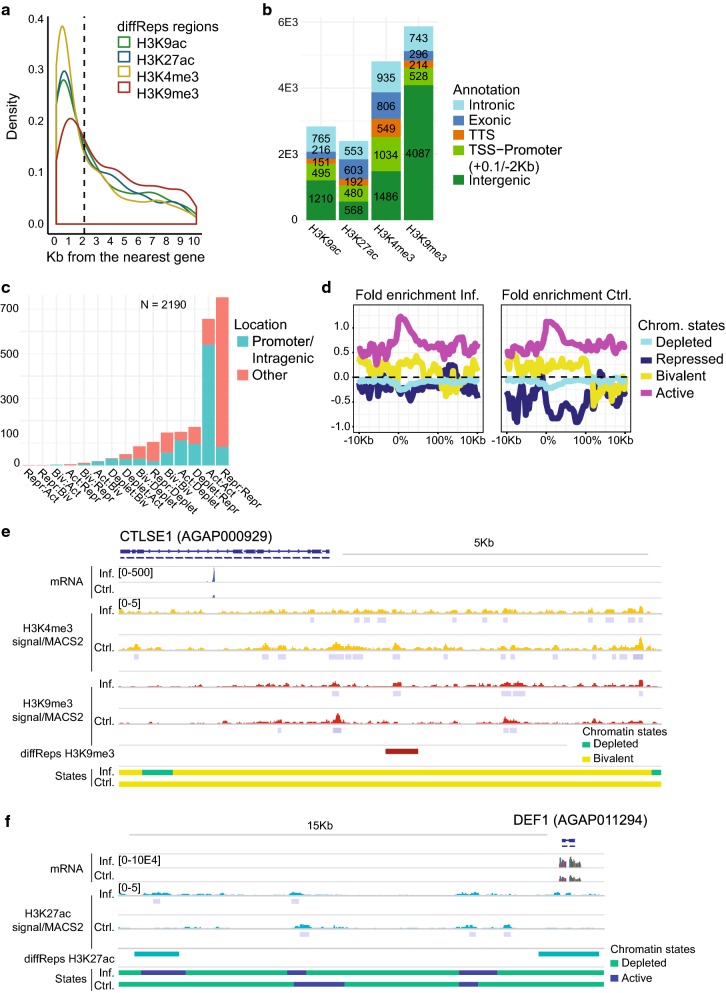
Changes in histone modification enrichment in response to infection. **a** Density plot showing the position (Kb upstream) of differential diffReps regions for each histone modification with respect to the ATG initiation codon of the nearest downstream gene. **b** Annotation of diffReps regions for each histone modification to genomic features: TSS-Promoters, TTSs, Intergenic, Intron and Exon regions. diffReps regions located − 2 Kb/+ 0.1 Kb from the ATG are annotated to TSS-Promoter regions. **c** Barplot showing the number and location of high-confidence diffReps regions and the chromatin state transitions between conditions associated with the region. **d** Profile plots showing predicted chromatin states in infected (left) and control (right) conditions at genes encoding for immune-related factors [34]. The graphs represent chromatin state fold enrichment (log(observed/expected)) with respect to the scaled gene bodies ± 10 Kb. **e**, **f** Histone enrichment profiles in the regions containing the CTLSE1 (AGAP000929) and DEF1 (AGAP011294) encoding genes. Tracks show normalized/input-corrected ChIP-seq signals and RNA-seq mapped read counts for each condition. The location of diffReps regions, MACS2 peaks and predicted chromatin states for each condition are included. All tracks are shown at equal scale

We find a considerable overlap in histone modification profiles between infected and uninfected mosquitoes, with more than 30,000 common ChIP-seq peaks between the two conditions. However, a portion of the peaks appears to be condition specific (8234 in infected and 14,138 in the uninfected) (Additional file 4: Figure S2C). Based on this observation, we used the diffReps software [33] to further investigate localized changes in hPTMs enrichment that might occur in response to *P. falciparum* infection. We identified 15,916 regions containing significantly different levels of ChIP-seq signal (*P* value < 10E−5) for all four histone modifications. The number of diffReps regions was similar for H3K9ac (2396) and H3K4me3 (2837), whereas H3K27ac and H3K9me3 displayed a larger number of differential regions (4810 and 5873, respectively) (Additional file 5: Table S4). Regions of differential active histone modifications between infected and control mosquitoes were primarily distributed near genes, upstream and downstream, or in introns. But they also occupied distal intergenic sites, particularly in the case of H3K9me3 (Fig. 2a, b). We applied a series of filtering thresholds (see Methods) to these differential regions to obtain a high-confidence set that we classified according to chromatin state transitions (ChromHMM segmentation) (Fig. 2c). In the majority of cases, the diffReps changes involved an enrichment or depletion in a certain histone modification without a chromatin state change, but we also reported chromatin state shifts between conditions: regions that were active upon infection or regions that were marked with active chromatin marks in control mosquito tissues and changed to depleted in the infected. There was also a considerable proportion of regions that switched between the depleted and H3K9me3-enriched states (Fig. 2c).

The Gene Ontology (GO) analysis shows that diffReps-annotated genes appeared significantly enriched in GO terms related to development, transcription regulation and metabolism (Additional file 5: Table S4). In addition to this analysis, we also looked for coincidences between the diffReps-annotated genes and genes that have been reported to be involved in the immune response to infection [34, 35]. Among diffReps-annotated genes, we found 133 genes encoding for proteins involved in the immune response (26 considering the high-confidence set of diffReps regions). Genes differing between conditions in their histone modification profiles encode proteins involved in apoptosis (IAP3 and IAP7), Clip-domain serine proteases and serine protease inhibitors (CLIPC, CLIPE, SRPN10 and SRPN4), C-type lectins (CTLs) (Fig. 2e), antimicrobial peptides (DEF1) (Fig. 2f), scavenger receptor (SRCR domain) with lysyl oxidase domain (SCRAL1) and components of Toll, NF and peptidoglycan recognition protein LC/immune deficiency (PGRP-LC/IMD) signaling pathways (Additional file 4: Figure S4A–C, Additional file 5: Table S4). Among the diffReps-annotated genes, we also find members of the mitogen-activated protein kinases (MAPKs) signaling pathway, such as ERK1/2 and MAP3K13, which have been recently studied in relation to mosquito JNK signaling and susceptibility to malaria infection [36]. The majority of these genes appeared enriched in the same chromatin states in both conditions but displayed a change in the relative abundance of active H3K4me3/K9ac/K27ac and/or the repressive H3K9me3 modification (Fig. 2d, Additional file 5: Table S4). There were only nine genes within the high-confidence set of diffReps-annotated genes that displayed differential chromatin state between conditions, and these include, for example, the long-caspase CASPL2-encoding gene and the fibrinogen-related protein-encoding gene (Additional file 4: Figure S4B, C, Additional file 5: Table S4).

### Complex relationship between chromatin and gene expression changes

**Fig. 3:**
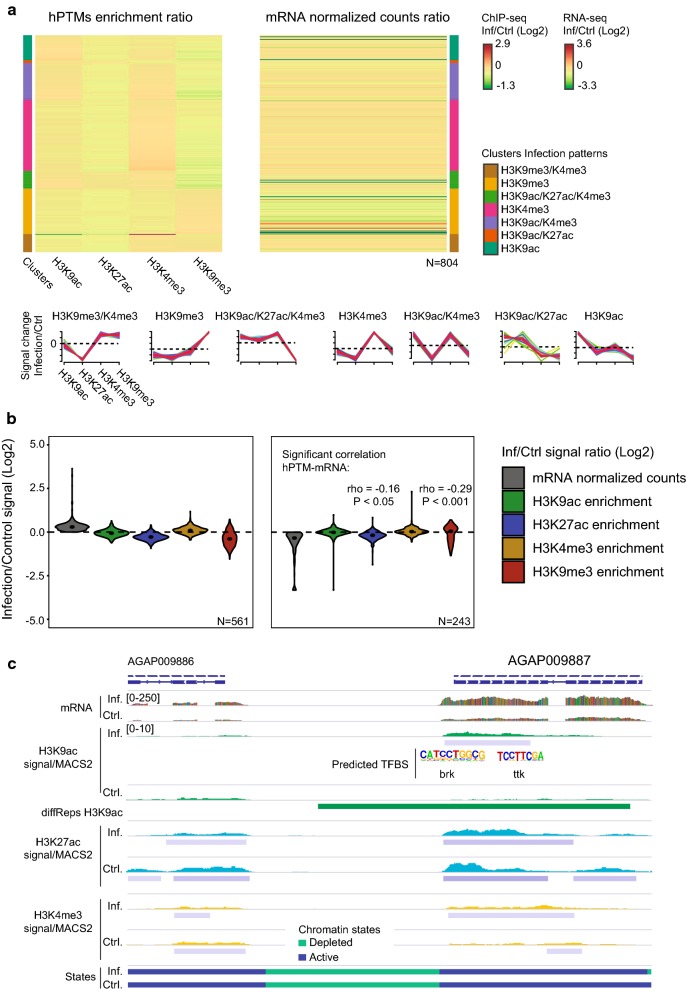
Association between histone modification differential enrichment and changes in gene expression. **a** Heatmaps showing clusters of genes (− 2 Kb) grouped by unique histone modification profiles identified in the soft clustering analysis (left) and corresponding changes in mRNA levels (right). ChIP-seq enrichment at the promoters and gene bodies is normalized (RPKM) and input-corrected. The signal corresponds to the ratio of ChIP-seq and mRNA levels in the infected versus the control condition. Data are log2-scaled and mean-centered. Representative profiles for each cluster showing various combinations of histone modification enrichment are included. All the profiles resulting from the soft clustering analysis are shown in Additional file 4: Figure S5. **b** Ratio of gene expression and histone modification enrichment between infected and control conditions for Mfuzz clusters more highly expressed in infected (left) and control (right) conditions. Data are the log2-scaled ratio between the infected and the control as in **a**. Spearman rank correlation coefficient (rho) and corresponding *P* value are shown for significant associations between histone modification enrichment and mRNA levels. **c** Histone enrichment profiles in the region containing the AGAP009887-encoding gene. Tracks show normalized/input-corrected ChIP-seq signals and RNA-seq mapped reads counts for each condition. The location of diffReps regions, MACS2 peaks, predicted transcription factor binding sites and predicted chromatin states for infected and control conditions are included. All tracks are shown at equal scale

In order to investigate the functional significance of the regions differentially enriched in various histone modifications, we selected high-confidence differential hPTMs regions that overlap gene bodies or are located up to 2 Kb upstream of genes. This analysis resulted in the identification of 1208 genes for all four hPTMs. We then applied a soft clustering approach using Mfuzz [37] to the − 2 Kb gene region. This analysis allowed us to group diffReps-annotated genes based on unique profiles of hPTM enrichment (Additional file 4: Figure S5) and to examine the correlation between changes in the histone modification patterns and the expression status. We found that genes differentially enriched in a condition in active histone marks (H3K27ac, H3K9ac and H3K4me3) tended to display high expression in that condition, whereas those that were marked with repressive H3K9me3 or bivalent H3K4me3/H3K9me3 tended to display low expression (Fig. 3a, Additional file 6: Table S5). However, when comparing the ratio of histone enrichment versus the ratio of gene expression values between infected and control mosquitoes, the correlation coefficient was low and non-significant, meaning that the infection condition influenced to a different extent chromatin and gene expression patterns. This was clearly shown when examining the ratio of enrichment of various hPTMs in the infected relative to the control, for infected expressed genes (left panel) or control expressed genes (right panel) (Fig. 3b). The ratio was in the same direction, above or below 1 for infected and control log2 values, respectively, only in the case of the H3K9me3. Despite complex patterns, this integrative analysis identified 278 genes in which the differential active or repressive histone modification enrichment coincided with a shift in gene expression between the infected and control condition (Fig. 3c, Additional file 4: Figure S4B, Additional file 6: Table S5).

**Fig. 4:**
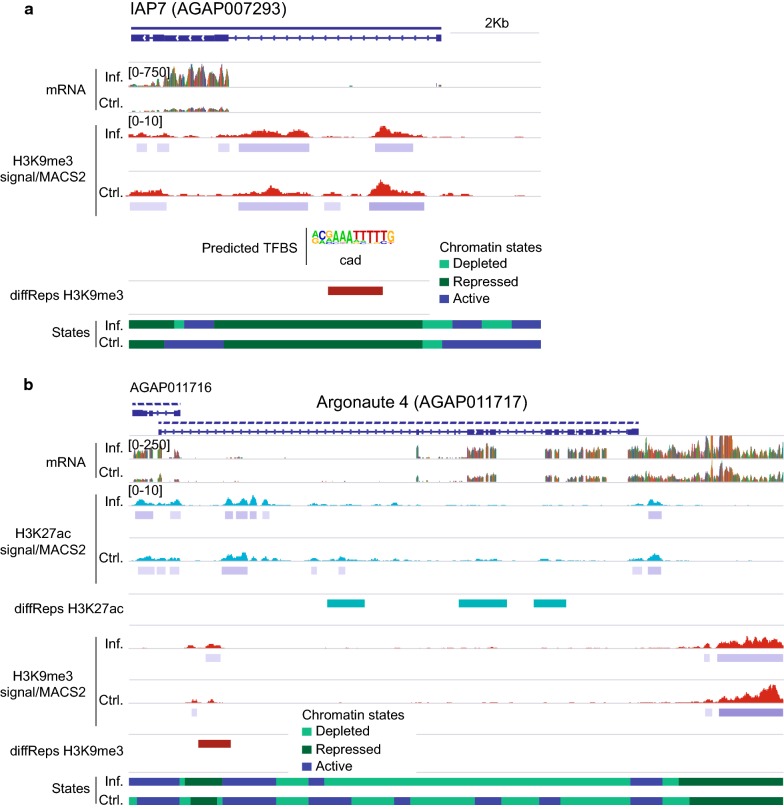
Significant differential gene expression and association with histone modifications differential enrichment. **a**, **b** Histone enrichment profiles in the regions containing the IAP7 (AGAP007293) and Argonaute 4 (AGAP011717) encoding genes. Tracks show normalized/input-corrected ChIP-seq signals and normalized RNA-seq mapped reads counts for each condition. The location of diffReps regions, MACS2 peaks, predicted transcription factor binding sites and predicted chromatin states for each condition are included. All tracks are shown at equal scale

In addition to the analysis of the diffReps-annotated genes, we also examined differentially expressed genes and looked for differential chromatin marks between conditions. The DESeq2 analysis on the RNA-seq data revealed 713 significant differentially expressed transcripts (*P* value < 0.05, 184 up-regulated genes and 529 down-regulated genes in the infected vs. the control condition) (Additional file 7: Table S6). We found 105 differential expressed genes that contain a diffReps region annotated to the promoter or the gene body. Of those, there were 72 in which the direction of the change (active or repressive histone marks) agreed with the functional prediction (up or down-regulation) (Additional file 7: Table S6). Same as above, the switch in the expression status was generally linked to changes in the H3K9me3 enrichment levels (Additional file 4: Figure S6). Examples of diffReps/DESeq2 genes were IAP7 (AGAP007293) and Argonaute 4 (AGAP011717). IAP7 was up-regulated in infected and H3K9me3 was depleted in this condition compared to the control (Fig. 4a), while AGAP011717 was expressed at higher levels in the control and this was associated with a gain in active histone modifications, mainly H3K27ac (Fig. 4b).

### Motif analysis of regions differentially enriched in histone modifications identifies transcription factor binding sites involved in transcriptional responses to infection

We conducted DNA-binding motif enrichment analysis on the set of ChIP-seq high-confidence diffReps regions that coincided with MACS2 peaks of significant enrichment, which includes 2018 peaks for all four histone modification marks (Additional file 5: Table S4). The goal of this approach applied to histone modification peaks is the discovery of unanticipated sequence motifs associated with specific histone marks, like transcription factor binding sites. This analysis revealed multiple motifs that are significantly enriched in sequences containing H3K9ac, H3K27ac, H3K4me3 and H3K9me3 differentially enriched peaks. Table 1 shows the list of novel motifs identified by HOMER on the set of diffReps regions for each histone modification and their similarities with known TF binding sites previously described in *Drosophila*. We found that the binding sites predicted at ChIP-seq peaks showed similarities with sequences bound by transcription factors such as Deaf1, pangolin (pan) and Dorsal (dl), linked to immunity gene expression regulation in *Drosophila*. We also found binding sites for several transcription factors associated with developmental functions, such as Caudal (cad), and reproduction, like Eip74EF (Table 1). In the set of diffReps regions that coincide with a MACS2 peak of enrichment, we computed the occurrences of predicted motifs (Additional file 5: Table S4). We identified eight immune genes, such as PGRPLA and IAP7, that contain one or multiple DNA motifs in the promoter or gene body (Fig. 4a). Only in a few cases, however, we could report that the gain in active histone modifications in the region that contains the motif is associated with a gene expression activation event. This is in part due to the fact that most genes contain multiple motifs, but also because the relationship between changes in histone modifications and gene expression patterns is not always in the same direction, as we noted in the previous section (Fig. 3a).

**Table 1:** List of consensus motifs corresponding to known transcription factors significantly enriched in the set of differential histone-modified regions that are associated with *P. falciparum* infection

Histone	Motif	*P* values	% Targets/background	TF BS predicted (score)	FlyBase record	Function
**H3K9ac**	CGTTCCCYWTTT	1E−23	18.40/1.06	**kni**/dmmpmm/fly (0.64)	http://flybase.org/reports/FBgn0001320	Development, gene expression
	AACKATTT	1E−23	18.40/1.02	**br-Z2**/dmmpmm/fly (0.727)	http://flybase.org/reports/FBgn0283451	Development, gene expression, reproduction, response to stimulus
	GTGCGTRA	1E−23	18.40/1.20	**z**/dmmpmm/fly (0.71)	http://flybase.org/reports/FBgn0000629.htm	Gene expression, nucleus
	GCGATAGA	1E−21	20.80/1.68	**Dref**/dmmpmm/fly (0.75)	http://flybase.org/reports/FBgn0015664.htm	Development, gene expression, metabolism
	MCCGAGCN	1E−20	16.00/0.64	**Deaf1**/dmmpmm/fly (0.70)	http://beta.flybase.org/reports/FBgn0013799.htm	Immune system, development, gene expression
	TAATCCRY	1E−19	16.00/0.93	**Gsc**/dmmpmm/fly (0.88)	http://flybase.org/reports/FBgn0010323	Gene expression, other molecular function
	CATCCTGGCG	1E−17	24.00/3.41	**brk**/dmmpmm/fly (0.679)	http://flybase.org/reports/FBgn0024250	Development, gene expression, signaling, response to stimulus
	TCCTTCGA	1E−15	17.60/2.23	**ttk**/dmmpmm/fly (0.71)	http://flybase.org/reports/FBgn0003870	Development, reproduction, response to stimulus, cell organization–biogenesis
	CTTGTTCTTC	1E−15	13.60/0.58	**pan**/dmmpmm/fly (0.63)	http://flybase.org/reports/FBgn0085432	Signaling, gene expression, immune system
	TTCGTAATAC	1E−15	13.60/1.46	**gt**/dmmpmm/fly (0.66)	http://flybase.org/reports/FBgn0001150	Development, gene expression, transcription factor
	GCTTGCTY	1E−14	16.80/2.34	**grh**/dmmpmm/fly (0.72)	http://flybase.org/reports/FBgn0259211	Development, biogenesis
	AGCTTTAA	1E−12	15.70/2.29	**zen**/dmmpmm/fly (0.70)	http://flybase.org/reports/FBgn0004053	Development, gene expression, transcription factor
**H3K27ac**	GGCTCGTC	1E−29	11.74/0.43	**h**/dmmpmm/fly(0.676)	http://flybase.org/reports/FBgn0001168.htm	Development, gene expression, DNA binding
	ATTTTTCCCC	1E−21	9.39/0.65	**dl**/dmmpmm/fly (0.69)	http://flybase.org/reports/FBgn0260632.htm	Immune system, development, gene expression
	AGAACAGTAA	1E−21	9.39/0.40	**ara**/dmmpmm/fly (0.67)	http://flybase.org/reports/FBgn0015904	Development, gene expression
	GTGCAGCTCG	1E−21	9.39/0.65	**sna**/dmmpmm/fly (0.65)	http://flybase.org/reports/FBgn0003448	Development, gene expression, DNA binding
	CAAAAACGCAAC	1E−21	9.39/0.67	**hb**/dmmpmm/fly (0.744)	http://flybase.org/reports/FBgn0001180	Development, gene expression, transcription factor
	CTATGTTT	1E−16	9.86/0.84	**pan**/dmmpmm/fly (0.80)	http://flybase.org/reports/FBgn0085432	Signaling, gene expression, immune system
	TCGATCGTCG	1E−15	7.51/0.00	**Dref**/dmmpmm/fly (0.60)	http://flybase.org/reports/FBgn0015664.html	Development, gene expression, DNA binding, transcription factor, cell cycle, DNA metabolism
	ATWTCTGC	1E−13	8.45/1.00	**grh**/dmmpmm/fly (0.714)	http://flybase.org/reports/FBgn0259211	Development, biogenesis
	CTTCADTGCGGA	1E−13	6.57/0.66	**slbo**/dmmpmm/fly (0.661)	http://flybase.org/reports/FBgn0005638	Development, gene expression, reproduction
	CACGAAGT	1E− 12	12.68/2.32	**Eip74EF**/dmmpmm/fly (0.772)	http://flybase.org/reports/FBgn0000567	Development, reproduction
**H3K4me3**	ARTTTTGTGT	1E−30	5.16/0.27	**br-Z3**/dmmpmm/fly (0.70)	http://flybase.org/reports/FBgn0283451	Development, gene expression, reproduction, response to stimuli
	TTTGATTCGTAA	1E−30	5.16/0.28	**ara**/dmmpmm/fly (0.67)	http://flybase.org/reports/FBgn0015904	Development, gene expression
	CTTCTTGCCCGA	1E−23	5.34/0.49	**Eip74EF**/dmmpmm/fly (0.62)	http://flybase.org/reports/FBgn0000567	Development, gene expression, reproduction
	CGAARAAGAR	1E−18	5.34/0.56	**pan**/dmmpmm/fly (0.70)	http://flybase.org/reports/FBgn0085432	Development, gene expression, response to stimulus, signaling, immune system
	TCCTCGTCGTTG	1E−16	7.55/1.60	**Aef1**/dmmpmm/fly (0.56)	http://flybase.org/reports/FBgn0005694.htm	Other molecular function
**H3K9me3**	AGCGCCTGGT	1E−26	8.82/0.61	**brk**/dmmpmm/fly (0.688)	http://flybase.org/reports/FBgn0024250	Development, gene expression, response to stimulus, signaling
	YCTGTGACCG	1E−24	8.46/0.59	**Hth**/dmmpmm/fly (0.72)	http://flybase.org/reports/FBgn0001235	Development, gene expression, DNA binding, transport/localization, transcription facto
	TCYKGWAKCKGA	1E−24	8.46/0.58	**Pnt**/dmmpmm/fly (0.60)	http://flybase.org/reports/FBgn0003118	Development, gene expression, response to stimulus, signaling, immune system, cell cycle/proliferation, DNA binding
	ACTCCAGATA	1E−21	7.72/0.57	**z**/dmmpmm/fly (0.61)	http://flybase.org/reports/FBgn0000629.html	Development, gene expression, protein metabolism, cell organization/biogenesis, enzyme
	CTAYTTAT	1E−21	7.72/0.18	**bin**/dmmpmm/fly (0.829)	http://flybase.org/reports/FBgn0045759	Development, gene expression, transcription factor
	ATCTCGGG	1E−21	7.72/0.53	**Deaf1**/dmmpmm/fly (0.68)	http://beta.flybase.org/reports/FBgn0013799.html	Development, gene expression, immune system, response to stimulus, transcription factor
	CATTCGAC	1E−21	9.56/0.81	**sd**/dmmpmm/fly (0.70)	http://flybase.org/reports/FBgn0003345	Development, gene expression, other molecular function, DNA binding, cell cycle/proliferation
	RCGAAATTTTTG	1E−20	7.35/0.22	**cad**/dmmpmm/fly (0.76)	http://flybase.org/reports/FBgn0000251	Development, gene expression, response to stimulus, transcription factor, DNA binding, immune system
	TTAGACGA	1E−20	7.35/0.40	**exd**/dmmpmm/fly (0.67)	http://flybase.org/reports/FBgn0000611	Development, gene expression, other molecular function, DNA binding, transcription factor

As a validation of our strategy to predict regulatory sites and TF binding, we reported the overlap between FAIRE-seq peaks determined in *A. gambiae* hemocytes in a previous study [27], with histone modifications ChIP-seq peaks in malaria-infected tissues. Even if the experimental conditions and cell types in the two studies are  not comparable, we found 9136 MACS2 ChIP-seq peaks and 2690 diffReps regions (10% and 17% of the total, respectively) that intersected with FAIRE-seq peaks.

These results collectively suggest that some of the transcription factors reported in our study could be involved in chromatin remodeling processes and the regulation of transcription of genes that are elicited in mosquitoes in response to *P. falciparum* infection.

## Discussion

Human malaria is a mosquito-borne disease responsible for around half-million deaths per year, *A. gambiae* being the main disease vector in Africa [1]. Infection by *P. falciparum* alters the phenotype and vector competence of mosquitoes with consequences for transmission and malaria epidemiology. However, the molecular players that regulate malaria infection-triggered responses are still poorly known. A considerable amount of work exists on the genomic basis of mosquito resistance to infection (reviewed by [38]), but there is a dearth of epigenomic studies on the relationship between chromatin and gene expression regulation in mosquitoes. In a previous study, we characterized for the first time genome-wide profiles of various histone modifications in *A. gambiae* and compared these patterns with chromatin maps published for *Drosophila* [26]. Here, we go a step further and perform an integrative analysis of ChIP-seq and RNA-seq data in the context of malaria infection in the natural conditions of transmission of the disease.

We report various chromatin states with links to functional gene expression in the study of global patterns of histone modifications in infected mosquitoes. H3K9ac/H3K4me3/H3K27ac histone marks are associated with the promoters of active genes, and repressive H3K9me3 is associated with silent genes. In agreement with previous studies in Drosophila, our results in mosquitoes show that gene structure is related to differences in the distribution of H3K27ac and H3K4me3 enrichment in the gene body, being the most common difference in the chromatin state of expressed genes that contain long introns relative to exons. We also report bivalent chromatin domains that have both repressive H3K9me3 and activating H3K4me3 histone marks in the same region. This pattern is typical of transcription factor genes that are expressed at low levels and have been also found associated with genes involved in development as well as gene imprinting [39, 40]. Despite the fact that the majority of peaks are present at promoters or gene bodies, we report a considerable number of ChIP-seq peaks (~ 40%) located more than 2 Kb from the nearest gene. It has been described that H3K27ac associates with active enhancers in *Drosophila* and other model organisms [41, 42]. In this study, we identified a number of distal H3K27ac-enriched regions that are depleted in H3K9ac/H3K4me3 promoter-like histone modifications and could correspond to enhancer-like sites. Future ChIP-seq studies using specific enhancer-mapping marks, such as H3K4me1, combined with chromatin accessibility profiling are required to confirm the presence of enhancer-like elements in mosquitoes.

Once we showed a relationship between chromatin states and function, our main purpose was to identify malaria-induced chromatin changes in mosquitoes. We could identify 15,916 histone-modified regions (2564 when considering high-confidence peaks) that appeared differentially methylated or acetylated upon infection. Various chromatin states (active, repressive and bivalent chromatin) were identified in the set of regions marked by differential levels of histone modifications. We also observed that differential regions generally display enrichment or depletion of individual marks at specific gene segments, but maintaining the same chromatin state. Importantly, there were 107 promoter or intragenic regions and 133 more distal sites (> 2 Kb) that correspond to genes involved in different pathways of mosquito innate immunity, either as activators or inhibitors of various responses (see [43, 44] for reviews). These include apoptosis, Clip-domain serine proteases and serine protease inhibitors, antimicrobial peptides, enzymes that catalyze generation and detoxification of reactive oxygen species, and components of Toll, NF and IMD immune signaling pathways.

The integration of regions differentially marked by histone modifications with expression levels of annotated genes resulted in the identification of 278 malaria-responsive genes. These genes show local differences in the enrichment of specific active/repressive histone marks that correlate with gene expression changes in the same direction. However, this is not the general rule and most of the differential regions by ChIP-seq do not display noticeable differences in gene expression, and the other way around for differentially expressed genes. It might be that there is a threshold enrichment level that is necessary to activate transcription. It is also unexpected that the comparison of infected with control tissues only identifies a few of these malaria-responsive genes corresponding to factors involved in the immune response. A possible explanation is that the majority of the immune response factors that have been described belong to the early innate immunity response that takes place between 2 and 24 h post-infection, at the ookinete stage, where the most part of the parasite recognition and killing occur [45, 46]. The samples analyzed in this study correspond to the oocyst stage, approximately six and seven days after an infective blood feeding, and the immune factors playing a role at this stage remain still poorly characterized [47].

Cis-regulatory elements are implicated in the control of gene expression because they contain specific DNA sequences that are binding sites for transcription factors and other chromatin remodelers, and often appear enriched in certain histone modifications. These elements are well mapped in *Drosophila* [48], but very few have been identified in mosquitoes [27, 49]. The analysis of differential ChIP-seq peaks located at promoters or gene bodies identified significant enrichment in binding sites that match consensus sequences of TFs previously described in *Drosophila*, including transcription factor deformed epidermal autoregulatory factor-1 (Deaf1) [50]. Indeed, Deaf1 is an important regulator of *Drosophila* immunity that could induce genes encoding for antimicrobial peptides [51]. Another example that we report in this study is Dorsal (Dl), a TF that functions downstream of the Toll pathway [50]. Finally, as a validation of our motif analysis, we find that a portion of the differential ChIP-peaks of histone modifications matches FAIRE-seq peaks described by a previous study on mosquito hemocytes [27]. In this study, authors reported that FAIRE sequences were enriched in binding sites for Deaf1, which is one of the top motifs reported in the present study. New approaches to profiling chromatin accessibility such as ATAC-seq will be useful to further characterize cis-regulatory sequences and TF binding in vivo.

## Conclusions

This study charts genomic landscapes of various active and repressive histone modifications in malaria-infected mosquitoes and integrates these profiles with RNA-seq data to quantify gene expression. Using this approach, we have identified malaria-responsive genes that display changes in the abundance of specific histone modifications. However, the relationship between chromatin and gene expression changes at differential regions is complex, and only a subset of genes shows correlated patterns that agree with the predicted function. Further research to identify regulatory sequences associated with these changes and the transcription factors with which they associate could provide new molecules and targets for vector control.

## Methods

### Mosquito rearing and experimental infections

Three- to 5-day-old female *A. gambiae* mosquitoes were sourced from an outbred colony established in 2008 and repeatedly replenished with F1 from wild-caught mosquito females collected in Soumousso, near Bobo-Dioulasso, southwestern Burkina Faso (West Africa). Mosquitoes were maintained under standard insectary conditions (27 ± 2 °C, 70 ± 5% relative humidity, 12:12 LD). Two independent experimental infections, biological replicates, were carried out by membrane blood feeding in the laboratory as described previously [52–55]. Briefly, females were fed through membranes on gametocyte-infected blood from malaria patients. Venous blood was collected and the serum was replaced by a non-immune AB serum to avoid transmission of human blocking factors. Dissection of mosquito midguts was performed in situ on adult females at 7 days post-blood meal. To determine infection levels, mosquito guts were stained with 2% mercurochrome before microscopic examination. Tissues were maintained in ice-cold Schneider’s insect culture medium (Sigma-Aldrich), and fresh tissues were immediately processed for chromatin and RNA analyses. Prevalence (percentage of infected mosquitoes) and intensity of infection (mean number of oocysts) for each infection performed are included in Additional file 1: Table S1.

### RNA isolation, library preparation and sequencing

We prepared RNA-seq libraries from RNA isolated from two biological replicates of uninfected and infected midgut samples. Total RNA was extracted from fresh mosquito tissues (~ 25 midguts) using the mirVana™ RNA Isolation Kit (Ambion^®^) according to the manufacturer protocol and used for mRNA library preparation. RNA concentration was quantified using a Qubit^®^ 2.0 Fluorometer, and RNA integrity was determined with an Agilent 2100 Bioanalyzer. Illumina libraries were prepared and sequenced at the HudsonAlpha Institute for Biotechnology, using an Illumina HiSeq 2000 sequencer, standard directional RNA-seq library construction and 50 bp paired end reads with ribosomal reduction (RiboMinus™ Eukaryote Kit, Ambion^®^).

### RNA-seq analysis

We mapped RNA paired directional reads to *A. gambiae* PEST strain genome version 4.3 publicly available at VectorBase [56] using TopHat v.2.0.13 [57]. We aligned reads using the option of library type set as first-strand for directional RNA-seq. We used SAMtools v.1.6 [58] for SAM and BAM file manipulation and conversion. We performed quality control analysis using QualiMap v.2.2.1 [59]. Statistics of the RNA-seq analysis for each condition and replicate are shown in Additional file 2: Table S2.

Quantification and differential gene expression analysis was conducted using HTSeq/DESeq2 packages [60, 61]. To count reads, HTSeq configuration parameters were set for a strand-specific assay to separate between sense and antisense transcripts. We used the matrix of raw reads counts as input for the DESeq2 R package, which performs library normalization and uses negative binomial generalized linear models to identify differentially expressed genes. The design included condition as main factor and infection as co-variable to control for inter-experiment variability. In this analysis, genes were considered significantly and differentially expressed if the *P* value was below 0.05.

Sets of differentially expressed genes between conditions, infected and control, were annotated based on the Gene Ontology (GO) and Kyoto Encyclopedia of Genes and Genomes (KEGG) using DAVID [62, 63].

### Chromatin immunoprecipitation

Chromatin immunoprecipitation in mosquito tissues was performed as previously described [26]. Antibodies to histone modifications used in this study were anti-H3K9ac (Millipore #07-352), anti-H3K4me3 (Abcam ab8580), anti-H3K27ac (Abcam ab4729) and anti-H3K9me3 (Abcam ab8898). ChIP-seq libraries were prepared following the procedure described by Bowman et al. [64] and using the HiFi Kapa Sybr library preparation kit (KapaBiosystems). To obtain the quantity needed to perform ChIP-seq, the two samples for which we have RNA-seq expression data were pooled, resulting in one biological replicate for infected mosquito midguts and one biological replicate of uninfected tissues. ChIP-seq libraries were sequenced at the HudsonAlpha Institute for Biotechnology using an Illumina HiSeq2000 sequencer.

### ChIP-seq data analysis

We performed quality control of Illumina reads using QualiMap v.2.2.1 [59] (Additional file 2: Table S2).

Correlation analysis was performed using deepTools2 (v.2.5.0) [65]. We reported Spearman rho correlation coefficients between each pair of histone modification datasets.

We mapped reads for various histone modifications and the input to *A. gambiae* PEST strain genome version 4.3 [56] using Bowtie v.2.2.9 [66] with default parameters except for –no-mixed. Reads were trimmed five bases from each read 5′ end (–trim5 5). Mapped reads were then sorted and deduplicated using SAMtools v.1.6. We applied a quality threshold of 10 in MAPQ score. All libraries were downsampled (SAMtools) to the same number of reads (9 M) for further downstream analysis. To calculate the enrichment, we used the BEDtools software suite (v.2.25.0) [67] to obtain the number of reads overlapping regions of interest. Resulting read counts were normalized (RPKM), input-corrected and log2-transformed using R. We conducted peak calling using the MACS2 (v.2.1.1) [30] “callpeak” module with -t and -c options and default parameters, except for -g 2.73E8 –keep-dup all -B –SPMR -q 0.01 –nomodel. We further assessed statistical significance of MACS2 peaks using the RECAP software [68] to recalibrate peak calling P-values. Over 99% of MACS2 peaks remained significant according to a recalibrated P-value threshold of 0.05 (Additional file 3: Table S3). For visualization in IGV [69, 70], tracks of input-corrected ChIP-seq signal were computed using the MACS2 “bdgcmp” module (-m ppois) on each pair of fragment pileup and control lambda bedGraph files from peak calling analysis [71].

To quantitatively compare histone modification profiles between infected and control mosquito tissues, we used the diffReps software [33]. This method uses a sliding window approach to identify regions that show significant changes in ChIP-seq signal, without constraining regions to compare by peak calling. ChIP-seq data for infected and control and the corresponding inputs were provided with -tr, -co, –btr and –bco options. Due to the lack of biological replicates, the statistical test used was G-test (-me gt). The threshold P-value was set to 10E−5. Other parameters were set as default except for –window 1000, as recommended for the scanning of histone modification peaks. We performed annotation of MACS2 peaks and diffReps regions to genomic features (TSSs, exons, introns and intergenic regions) using the annotatePeaks.pl module in HOMER (v.3.12) [72]. Based on the density distribution of the distances from upstream MACS2 peaks and diffReps regions to the nearest ATG site, we considered 2 Kb upstream from the translation start site ATG as the putative promoter region.

We used the chromatin state segmentation software ChromHMM [31] to compute genome-wide chromatin state predictions in each condition based on relative enrichment levels of histone modifications. For the binarization of the genome, we used default parameters except for -b 200. We chose a four-states model assuming chromatin states with high levels of enrichment of each histone modification. The chromatin states we found were: depleted (low levels of enrichment for all histone modifications), high levels of enrichment for H3K9me3, high levels of enrichment for H3K9me3 and H3K4me3 (bivalent state) and high levels of enrichment for H3K9ac, H3K27ac and H3K4me3. According to the ChromHMM segmentation, most of the genome is in a depleted state. We assigned predicted chromatin states to different features, such as MACS2 peaks and diffReps regions, using the intersect tool from BEDtools, and we required a minimum overlap between the regions of 51% (-f 0.51).

To obtain a high-confidence set of diffReps regions, we applied a filtering based on multiple thresholds. We filtered out those diffReps regions located in depleted chromatin states (ChromHMM segmentation) at each corresponding condition and displaying FDR > 0.05. We also divided regions in three quantile groups (cut2 function in the Hmisc R package) according to their mean values in log2 Fold Change and in average normalized counts and fold enrichment versus input at each corresponding condition. Regions that fall in the lower quantiles were discarded (Additional file 5: Table S4).

We performed Gene Ontology (GO) terms overrepresentation tests for the sets of genes of interest annotated to diffReps regions using PANTHER Overrepresentation Test [73, 74]. We chose Fisher’s exact with FDR multiple tests correction and applied a threshold of FDR < 0.05. Sets of differentially expressed genes between conditions, infected and control, were annotated based on the Gene Ontology and Kyoto Encyclopedia of Genes and Genomes (KEGG) using DAVID. We used ChromHMM segmentation and plotEnrichment function in chromstaR R package [75] to assess enrichment of predicted chromatin states in certain features of interest, e.g., the subset of genes encoding for immune response factors. Average profile plots and heatmaps representing enrichment of histone modifications (RPKM normalized, input-corrected and centered on gene coordinates) were built using ngs.plot (v.2.61) [76].

### Integration of RNA and ChIP-seq data

To connect patterns of histone modifications with regulation of gene expression, we ordered genes annotated to MACS2 peaks by mRNA levels and showed histone modification enrichment levels at those gene bodies and promoter regions. Correlation between histone modification enrichment levels and mRNA levels was assessed using Spearman rank correlation test (cor.test R function). To measure the quantitative association between histone modifications and mRNA levels, we fitted a linear regression model using the lm R function [77–79]. The model considered mRNA levels as response and histone modification enrichment levels as covariates and computed the R-squared (R2) value, which measures the proportion of the variance of the response that is explained by changes in the covariates. For selecting the best model and testing the linear model fit of different combinations of histone modifications, we used the MuMIn R package (dredge function) [80] and kept the model with higher likelihood and R2 and delta AICc < 2. Multicollinearity was assessed using the R package car [81]. VIF values for all the covariates were below 3. For assessing the relative importance of the covariates as predictors, we used the R2 decomposition method implemented in the calc.relimp function of the relaimpo R package [82].

To connect differential enrichment of histone modifications with regulation of gene expression, we filtered those genes containing high-confidence diffReps regions in promoters and gene bodies and performed a soft clustering approach using the Mfuzz R package [37] over the ratio of histone modifications between conditions (ratio of enrichment at infected to uninfected samples). Using a standard m fuzzy c-means parameter of 1.7, a total of 30 clusters were created. Clusters with certain patterns of histone modifications were isolated creating unique groups (Additional file 4: Figure S5). Only elements with a membership value higher than 51% within each particular cluster were considered. Next, we used the clustering order based on the ratio of histone modification enrichment to show mRNA levels of the corresponding genes (ratio of mRNA levels at infected to uninfected samples). To check the validity of our results and to further assess the functional output and transcriptional shift associated with different chromatin states, we focused on patterns showing maximum enrichment of certain histone modification at both infected and uninfected. We categorized genes into high, medium or low expression groups at each condition by dividing the mRNA levels in three quantile groups according to their means (cut2 R function), and we filtered out low-expressed genes. Based on the soft clustering of each region and each gene mRNA level, we then isolated those cases where differential histone enrichment profiles, a gain/loss in active hPTMs or gain/loss in the repressive H3K9me3 modification, coincide with the expected functional output: up- or down-regulation of the gene. We also performed a soft clustering analysis with the same parameters as above computing histone modification enrichment ratios at promoters and gene bodies of significant differentially expressed genes according to DESeq2 (*P* value < 0.05), with similar results (Additional file 4: Figures S7, S8, Additional file 8: Table S7).

Heatmaps showing histone modification enrichment and mRNA levels were built using the iheatmapr R package [83]. Bar and violin plots were produced using the ggplot2 R package [84]. For comparative and visualization purposes, histone enrichment signals and mRNA levels were log2-transformed. When computing histone modifications enrichments and ratios, a pseudocount (0.1) was added to obtain finite values (avoid dividing by 0 in ratios) when input-correcting or converting the signal to log2 scale. When categorizing values in quantile groups according to their means, high, medium and low groups, we used the cut2 function in the Hmisc R package [85].

We identified potential enhancer-like regions in infected and control conditions by taking distal (> 2 Kb upstream from the ATG) H3K27ac MACS2 peaks overlapping with active chromatin state regions according to the ChromHMM genome segmentation analysis. From these active regions, we subset only those overlapping with H3K27ac MACS2 peaks of enrichment but that appeared depleted in all other histone modifications. Average profile plots representing RNA-seq signal (RPM) at these regions were built using ngs.plot (v.2.61).

### Motif analysis

We conducted de novo motif analysis using HOMER software (v.3.12) [72] on the set of MACS2 peaks that intersect with high-confidence diffReps regions and annotate to genes (promoters and gene bodies). For this analysis, we considered the center of the ChIP-seq peak region and slopped 100 bp in each direction. We limited the number of background sequences to double the number of ChIP-seq target sequences for each histone modification. Only motifs enriched in more than 5% of the target sequences and below a threshold P-value of 10E−15 were considered, and results corresponding to low complexity motifs and offsets or degenerate versions of highly enriched motifs were avoided. The purpose of this analysis was to identify enrichment in particular sequence footprints associated with changes in histone modifications occupancy that are associated with *P. falciparum* infection. We used the annotatePeaks.pl module in HOMER to find motif occurrences in each histone-specific ChIP-seq peaks sets.

## Additional files

**Additional file 1: Table S1.** Information on the prevalence of infection (percentage of infected mosquitoes) and the intensity of infection (mean number of oocysts).

**Additional file 2: Table S2.** Summary of RNA-seq and ChIP-seq alignment statistics.

**Additional file 3: Table S3.** Set of MACS2 ChIP-seq peaks for each histone modification in the infected and uninfected mosquito tissues. Information on the annotation to genomic features, location, Gene Ontology (GO) terms and data from PFAM and KEGG databases is included.

**Additional file 4.** Supplementary figures 1–8.

**Additional file 5: Table S4.** Set of regions with differential enrichment of histone modifications between infected and uninfected mosquito tissues. Information on the annotation to genomic features, location, predicted chromatin states, MACS2 peaks overlap, mRNA levels of the nearest gene, GO terms and data from PFAM and KEGG databases is included.

**Additional file 6: Table S5.** Results of the soft clustering analysis on the set of genes with high-confidence diffReps regions located in the promoter or the gene body. Genes are clustered based on the histone modification enrichment profiles (ratio of enrichment in infected versus control condition). The ratio is normalized (RPKM) and input-corrected. The ChIP-seq signal for each histone modification and mRNA levels of the corresponding genes are included.

**Additional file 7: Table S6.** Set of genes showing significant differential gene expression according to DESeq2 analysis. Information on GO terms and PFAM and KEGG database annotations are included. Overlap with high-confidence diffReps regions is indicated.

**Additional file 8: Table S7.** Results of the soft clustering analysis on the set of significant differentially expressed genes according to DESeq2 analysis. Genes are clustered based on the histone modification enrichment profiles (ratio of enrichment in infected versus control condition). The ratio is normalized (RPKM) and input-corrected. The ChIP-seq signal for each histone modification and mRNA levels of the corresponding genes are included.

## References

[1] WHO (2017). World malaria report.

[2] Vlachou D, Schlegelmilch T, Christophides GK, Kafatos FC (2005). Functional genomic analysis of midgut epithelial responses in Anopheles during Plasmodium invasion. Curr Biol.

[3] Blandin S, Levashina EA (2004). Mosquito immune responses against malaria parasites. Curr Opin Immunol.

[4] Akman-Anderson L, Olivier M, Luckhart S (2007). Induction of nitric oxide synthase and activation of signaling proteins in Anopheles mosquitoes by the malaria pigment, hemozoin. Infect Immun.

[5] Blandin SA, Marois E, Levashina EA (2008). Antimalarial responses in *Anopheles gambiae*: from a complement-like protein to a complement-like pathway. Cell Host Microbe.

[6] Bartholomay LC, Michel K (2018). Mosquito immunobiology: the intersection of vector health and vector competence. Ann Rev Entomol.

[7] Pollitt LC, Bram JT, Blanford S, Jones MJ, Read AF (2015). Existing infection facilitates establishment and density of malaria parasites in their mosquito vector. PLoS Pathog.

[8] Simões ML, Dimopoulos G (2015). A mosquito mediator of parasite-induced immune priming. Trends Parasitol.

[9] Lambrechts L, Chavatte JM, Snounou G, Koella JC (2006). Environmental influence on the genetic basis of mosquito resistance to malaria parasites. Proc Biol Sci.

[10] Lefevre T, Vantaux A, Dabire KR, Mouline K, Cohuet A (2013). Non-genetic determinants of mosquito competence for malaria parasites. PLoS Pathog.

[11] Feil R, Fraga MF (2012). Epigenetics and the environment: emerging patterns and implications. Nat Rev Genet.

[12] Gut P, Verdin E (2013). The nexus of chromatin regulation and intermediary metabolism. Nature.

[13] Kouzarides T (2007). Chromatin modifications and their function. Cell.

[14] Yin H, Sweeney S, Raha D, Snyder M, Lin H (2011). A high-resolution whole-genome map of key chromatin modifications in the adult *Drosophila melanogaster*. PLoS Genet.

[15] Merkling SH, Bronkhorst AW, Kramer JM, Overheul GJ, Schenck A, Van Rij RP (2015). The epigenetic regulator G9a mediates tolerance to RNA virus infection in Drosophila. PLoS Pathog.

[16] Anreiter I, Kramer JM, Sokolowski MB (2017). Epigenetic mechanisms modulate differences in Drosophila foraging behavior. Proc Natl Acad Sci USA.

[17] Dobson AJ, Ezcurra M, Flanagan CE, Summerfield AC, Piper MDW, Gems D, Alic N (2017). Nutritional programming of Lifespan by FOXO inhibition on sugar-rich diets. Cell Rep.

[18] Bantignies F, Grimaud C, Lavrov S, Gabut M, Cavalli G (2003). Inheritance of Polycomb-dependent chromosomal interactions in Drosophila. Genes Dev.

[19] Margueron R, Reinberg D (2010). Chromatin structure and the inheritance of epigenetic information. Nat Rev Genet.

[20] Campos EI, Stafford JM, Reinberg D (2014). Epigenetic inheritance: histone bookmarks across generations. Trends Cell Biol.

[21] Stroud H, Su SC, Hrvatin S, Greben AW, Renthal W, Boxer LD, Nagy MA, Hochbaum, Kinde B, Gabel HW (2017). Early-life gene expression in neurons modulates lasting epigenetic states. Cell.

[22] Coleman RT, Struhl G (2017). Causal role for inheritance of H3K27me3 in maintaining the OFF state of a Drosophila HOX gene. Science.

[23] Osborne AJ, Dearden PK (2017). A ‘phenotypic hangover’: the predictive adaptive response and multigenerational effects of altered nutrition on the transcriptome of *Drosophila melanogaster*. Environ Epigenet.

[24] Ciabrelli F, Comoglio F, Fellous S, Bonev B, Ninova M, Szabo Q, Xuéreb A, Klopp C, Aravin A, Paro R (2017). Stable Polycomb-dependent transgenerational inheritance of chromatin states in Drosophila. Nat Genet.

[25] Gomez-Diaz E, Jorda M, Peinado MA, Rivero A (2012). Epigenetics of host-pathogen interactions: the road ahead and the road behind. PLoS Pathog.

[26] Gómez-Díaz E, Rivero A, Chandre F, Corces VG (2014). Insights into the epigenomic landscape of the human malaria vector *Anopheles gambiae*. Front Genet.

[27] Pérez-Zamorano B, Rosas-Madrigal S, Lozano OAM, Méndez MC, Valverde-Garduño V (2017). Identification of cis-regulatory sequences reveals potential participation of lola and Deaf1 transcription factors in *Anopheles gambiae* innate immune response. PLoS ONE.

[28] Zakovic S, Levashina EA (2017). NF-κB-like signaling pathway REL2 in immune defenses of the Malaria vector *Anopheles gambiae*. Front Cell Infect Microbiol.

[29] Tripet F, Aboagye-Antwi F, Hurd H (2008). Ecological immunology of mosquito–malaria interactions. Trends Parasitol.

[30] Zhang Y, Liu T, Meyer CA, Eeckhoute J, Johnson DS, Bernstein BE, Nusbaum C, Myers RM, Brown M, Li W (2008). Model-based analysis of ChIP-Seq (MACS). Genome Biol.

[31] Ernst J, Kellis M (2012). ChromHMM: automating chromatin-state discovery and characterization. Nat Methods.

[32] Kharchenko PV, Alekseyenko AA, Schwartz YB, Minoda A, Riddle NC, Ernst J, Sabo PJ, Larschan E, Gorchakov AA, Gu T (2011). Comprehensive analysis of the chromatin landscape in *Drosophila melanogaster*. Nature.

[33] Shen L, Shao N-Y, Liu X, Maze I, Feng J, Nestler EJ (2013). diffReps: detecting differential chromatin modification sites from ChIP-seq data with biological replicates. PLoS ONE.

[34] Brucker RM, Funkhouser LJ, Setia S, Pauly R, Bordenstein SR (2012). Insect innate immunity database (IIID): an annotation tool for identifying immune genes in insect genomes. PLoS ONE.

[35] Waterhouse RM, Kriventseva EV, Meister S, Xi Z, Alvarez KS, Bartholomay LC, Barillas-Mury C, Bian G, Blandin S, Christensen BM (2007). Evolutionary dynamics of immune-related genes and pathways in disease-vector mosquitoes. Science.

[36] Souvannaseng L, Hun LV, Baker H, Klyver JM, Wang B, Pakpour N, Bridgewater JM, Napoli E, Giulivi C, Riehle MA (2018). Inhibition of JNK signaling in the Asian malaria vector *Anopheles stephensi* extends mosquito longevity and improves resistance to *Plasmodium falciparum* infection. PLoS Pathog.

[37] Kumar L, Futschik ME (2007). Mfuzz: a software package for soft clustering of microarray data. Bioinformation.

[38] Rinker DC, Pitts RJ, Zwiebel LJ (2016). Disease vectors in the era of next generation sequencing. Genome Biol.

[39] Bernstein BE, Mikkelsen TS, Xie X, Kamal M, Huebert DJ, Cuff J, Fry B, Meissner A, Wernig M, Plath K (2006). A bivalent chromatin structure marks key developmental genes in embryonic stem cells. Cell.

[40] Voigt P, Tee WW, Reinberg D (2013). A double take on bivalent promoters. Genes Dev.

[41] Creyghton MP, Cheng AW, Welstead GG, Kooistra T, Carey BW, Steine EJ, Hanna J, Lodato MA, Frampton GM, Sharp PA (2010). Histone H3K27ac separates active from poised enhancers and predicts developmental state. Proc Natl Acad Sci USA.

[42] Pradeepa MM (2017). Causal role of histone acetylations in enhancer function. Transcription.

[43] Lemaitre B, Hoffmann J (2007). The host defense of *Drosophila melanogaster*. Annu Rev Immunol.

[44] Marois E (2011). The multifaceted mosquito anti-Plasmodium response. Curr Opin Microbiol.

[45] Habtewold T, Groom Z, Christophides GK (2017). Immune resistance and tolerance strategies in malaria vector and non-vector mosquitoes. Parasites Vectors.

[46] Baton LA, Ranford-Cartwright LC (2012). Ookinete destruction within the mosquito midgut lumen explains *Anopheles albimanus* refractoriness to *Plasmodium falciparum* (3D7A) oocyst infection. Int J Parasitol.

[47] Smith RC, Barillas-Mury C (2016). *Plasmodium oocysts*: overlooked targets of mosquito immunity. Trends Parasitol.

[48] Negre N, Brown CD, Ma L, Bristow CA, Miller SW, Wagner U, Kheradpour P, Eaton ML, Loriaux P, Sealfon R (2011). A cis-regulatory map of the *Drosophila genome*. Nature.

[49] Behura SK, Sarro J, Li P, Mysore K, Severson DW, Emrich SJ, Duman-Scheel M (2016). High-throughput cis-regulatory element discovery in the vector mosquito *Aedes aegypti*. BMC Genom.

[50] Govind S (2008). Innate immunity in Drosophila: pathogens and pathways. Insect Sci.

[51] Reed DE, Huang XM, Wohlschlegel JA, Levine MS, Senger K (2008). DEAF-1 regulates immunity gene expression in Drosophila. Proc Natl Acad Sci USA.

[52] Alout H, Djègbè I, Chandre F, Djogbénou LS, Dabiré RK, Corbel V, Cohuet A (2014). Insecticide exposure impacts vector–parasite interactions in insecticide-resistant malaria vectors. Proc R Soc B.

[53] Vantaux A, Dabiré KR, Cohuet A, Lefèvre T (2014). A heavy legacy: offspring of malaria-infected mosquitoes show reduced disease resistance. Malar J.

[54] Da DF, Churcher TS, Yerbanga RS, Yaméogo B, Sangaré I, Ouedraogo JB, Sinden RE, Blagborough AM, Cohuet A (2015). Experimental study of the relationship between Plasmodium gametocyte density and infection success in mosquitoes; implications for the evaluation of malaria transmission-reducing interventions. Exp Parasitol.

[55] Gendrin M, Rodgers FH, Yerbanga RS, Ouédraogo JB, Basáñez M-G, Cohuet A, Christophides GK (2015). Antibiotics in ingested human blood affect the mosquito microbiota and capacity to transmit malaria. Nat Commun.

[56] Giraldo-Calderon GI, Emrich SJ, MacCallum RM, Maslen G, Dialynas E, Topalis P, Ho N, Gesing S, VectorBase C, Madey G (2015). VectorBase: an updated bioinformatics resource for invertebrate vectors and other organisms related with human diseases. Nucleic Acids Res.

[57] Kim D, Pertea G, Trapnell C, Pimentel H, Kelley R, Salzberg SL (2013). TopHat2: accurate alignment of transcriptomes in the presence of insertions, deletions and gene fusions. Genome Biol.

[58] Li H, Handsaker B, Wysoker A, Fennell T, Ruan J, Homer N, Marth G, Abecasis G, Durbin R (2009). Genome project data processing S: the sequence alignment/map format and SAMtools. Bioinformatics.

[59] Okonechnikov K, Conesa A, Garcia-Alcalde F (2016). Qualimap 2: advanced multi-sample quality control for high-throughput sequencing data. Bioinformatics.

[60] Anders S, Pyl PT, Huber W (2015). HTSeq—a python framework to work with high-throughput sequencing data. Bioinformatics.

[61] Love MI, Huber W, Anders S (2014). Moderated estimation of fold change and dispersion for RNA-seq data with DESeq2. Genome Biol.

[62] Jiao X, Sherman BT, Huang DW, Stephens R, Baseler MW, Lane HC, Lempicki RA (2012). DAVID-WS: a stateful web service to facilitate gene/protein list analysis. Bioinformatics.

[63] Huang DW, Sherman BT, Lempicki RA (2008). Systematic and integrative analysis of large gene lists using DAVID bioinformatics resources. Nat Protoc.

[64] Bowman SK, Simon MD, Deaton AM, Tolstorukov M, Borowsky ML, Kingston RE (2013). Multiplexed Illumina sequencing libraries from picogram quantities of DNA. BMC Genom.

[65] Ramirez F, Ryan DP, Gruning B, Bhardwaj V, Kilpert F, Richter AS, Heyne S, Dundar F, Manke T (2016). deepTools2: a next generation web server for deep-sequencing data analysis. Nucleic Acids Res.

[66] Langmead B, Salzberg SL (2012). Fast gapped-read alignment with Bowtie 2. Nat Methods.

[67] Quinlan AR, Hall IM (2010). BEDTools: a flexible suite of utilities for comparing genomic features. Bioinformatics.

[68] Chitpin JG (2018). Awdeh A.

[69] Robinson JT, Thorvaldsdottir H, Winckler W, Guttman M, Lander ES, Getz G, Mesirov JP (2011). Integrative genomics viewer. Nat Biotechnol.

[70] Thorvaldsdottir H, Robinson JT, Mesirov JP (2013). Integrative genomics viewer (IGV): high-performance genomics data visualization and exploration. Brief Bioinform.

[71] MACS. Build signal track. https://github.com/taoliu/MACS/wiki/Build-Signal-Track.

[72] Heinz S, Benner C, Spann N, Bertolino E, Lin YC, Laslo P, Cheng JX, Murre C, Singh H, Glass CK (2010). Simple combinations of lineage-determining transcription factors prime cis-regulatory elements required for macrophage and B cell identities. Mol Cell.

[73] Thomas PD, Campbell MJ, Kejariwal A, Mi H, Karlak B, Daverman R, Diemer K, Muruganujan A, Narechania A (2003). PANTHER: a library of protein families and subfamilies indexed by function. Genome Res.

[74] Mi H, Dong Q, Muruganujan A, Gaudet P, Lewis S, Thomas PD (2010). PANTHER version 7: improved phylogenetic trees, orthologs and collaboration with the gene ontology consortium. Nucleic Acids Res.

[75] Taudt A, Nguyen MA, Heinig M, Johannes F, Colome-Tatche M (2016). chromstaR: tracking combinatorial chromatin state dynamics in space and time. bioRxiv.

[76] Shen L, Shao N, Liu X, Nestler E (2014). ngs.plot: quick mining and visualization of next-generation sequencing data by integrating genomic databases. BMC Genom.

[77] Gomez-Diaz E, Yerbanga RS, Lefevre T, Cohuet A, Rowley MJ, Ouedraogo JB, Corces VG (2017). Epigenetic regulation of *Plasmodium falciparum* clonally variant gene expression during development in *Anopheles gambiae*. Sci Rep.

[78] Zhou X, Cain CE, Myrthil M, Lewellen N, Michelini K, Davenport ER, Stephens M, Pritchard JK, Gilad Y (2014). Epigenetic modifications are associated with inter-species gene expression variation in primates. Genome Biol.

[79] Dong X, Greven MC, Kundaje A, Djebali S, Brown JB, Cheng C, Gingeras TR, Gerstein M, Guigó R, Birney E (2012). Modeling gene expression using chromatin features in various cellular contexts. Genome Biol.

[80] Bartoń K. Multi-model inference. R package version. 2018; 1: 42.

[81] Fox J, Weisberg S (2018). An R companion to applied regression.

[82] Grömping U (2006). Relative importance for linear regression in R: the package relaimpo. J Stat Softw.

[83] Schep AN, Kummerfeld SK (2017). iheatmapr: interactive complex heatmaps in R. J Open Sour Softw.

[84] Wickham H (2016). ggplot2: elegant graphics for data analysis.

[85] Harrell FE with contributions from Charles Dupont and many others. Hmisc: Harrell miscellaneous. R package version. 2017; 4:0–3.

