# Supplementary material for: Chromatin changes in Anopheles gambiae induced by Plasmodium falciparum infection
Source: Epigenetics Chromatin. 2019 Jan 7;12:5. doi: 10.1186/s13072-018-0250-9 (PMC6322293; doi:10.1186/s13072-018-0250-9)
Supplement: Supplementary file 4 — Additional file 4. Supplementary figures 1–8. [file 13072_2018_250_MOESM4_ESM.pdf]

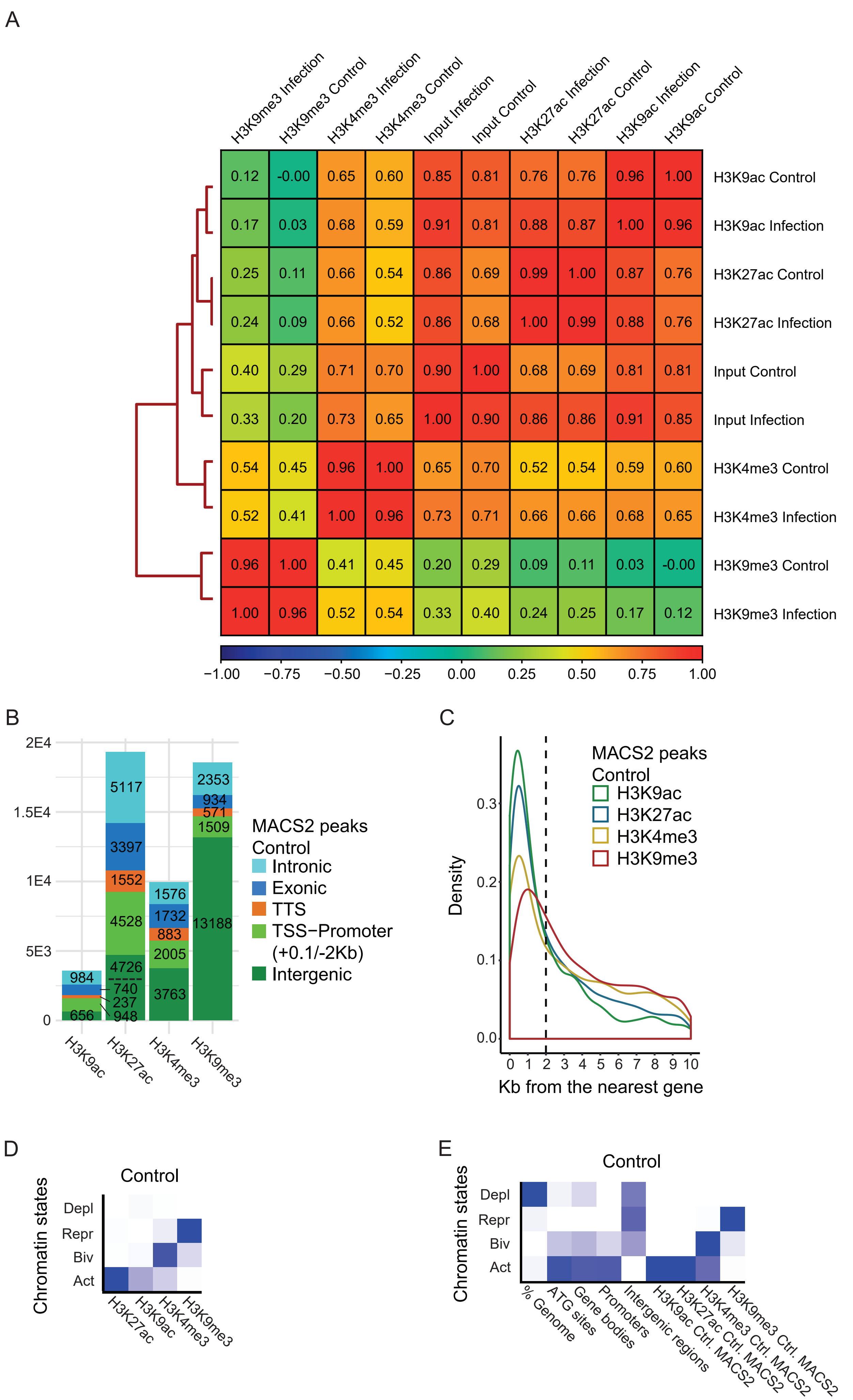

A
H3K9me3 Infection
H3K9me3 Control
H3K4me3 Infection
H3K4me3 Control
Input Infection
Input Control
H3K27ac Infection
H3K27ac Control
H3K9ac Infection
H3K9ac Control
0.12 -0.00 0.65 0.60 0.85 0.81 0.76 0.76 0.96 1.00 H3K9ac Control
0.17 0.03 0.68 0.59 0.91 0.81 0.88 0.87 1.00 0.96 H3K9ac Infection
0.25 0.11 0.66 0.54 0.86 0.69 0.99 1.00 0.87 0.76 H3K27ac Control
0.24 0.09 0.66 0.52 0.86 0.68 1.00 0.99 0.88 0.76 H3K27ac Infection
0.40 0.29 0.71 0.70 0.90 1.00 0.68 0.69 0.81 0.81 Input Control
0.33 0.20 0.73 0.65 1.00 0.90 0.86 0.86 0.91 0.85 Input Infection
0.54 0.45 0.96 1.00 0.65 0.70 0.52 0.54 0.59 0.60 H3K4me3 Control
0.52 0.41 1.00 0.96 0.73 0.71 0.66 0.66 0.68 0.65 H3K4me3 Infection
0.96 1.00 0.41 0.45 0.20 0.29 0.09 0.11 0.03 -0.00 H3K9me3 Control
1.00 0.96 0.52 0.54 0.33 0.40 0.24 0.25 0.17 0.12 H3K9me3 Infection
−1.00 −0.75 −0.50 −0.25 0.00 0.25 0.50 0.75 1.00
B
2E4
1.5E4
1E4
5E3
0
984
656
5117
3397
1552
4528
4726
740
237
948
1576
1732
883
2005
3763
2353
934
571
1509
13188
H3K9ac
H3K27ac
H3K4me3
H3K9me3
MACS2 peaks
Control
Intronic
Exonic
TTS
TSS−Promoter
(+0.1/-2Kb)
Intergenic
C
MACS2 peaks
Control
H3K9ac
H3K27ac
H3K4me3
H3K9me3
Density
0.3
0.2
0.1
0.0
0 1 2 3 4 5 6 7 8 9 10
Kb from the nearest gene
D
Control
Chromatin states
Depl
Repr
Biv
Act
H3K27ac
H3K9ac
H3K4me3
H3K9me3
E
Control
Chromatin states
Depl
Repr
Biv
Act
% Genome
ATG sites
Gene bodies
Promoters
Intergenic regions
H3K9ac Ctrl. MACS2
H3K27ac Ctrl. MACS2
H3K4me3 Ctrl. MACS2
H3K9me3 Ctrl. MACS2

Figure S1

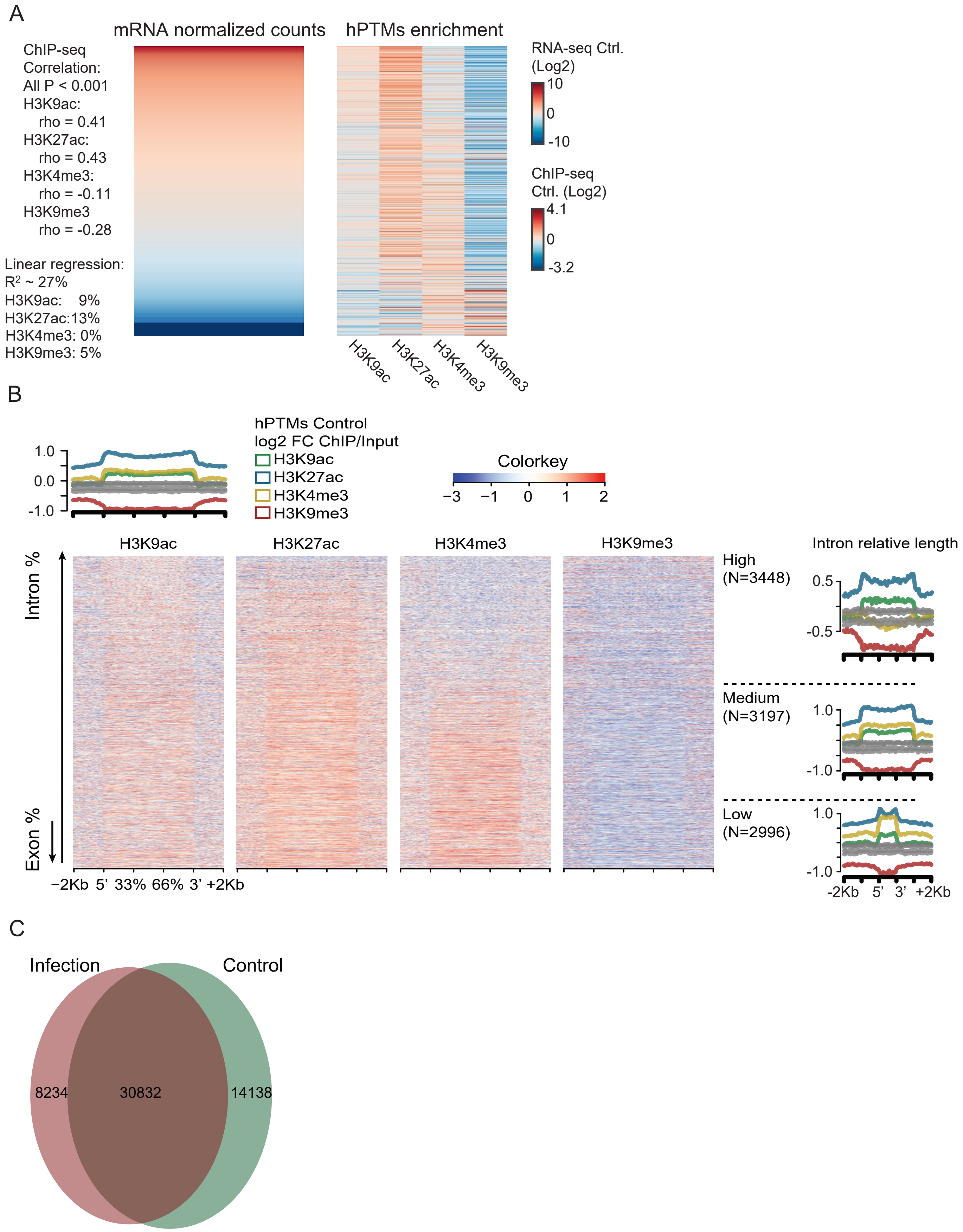

A
mRNA normalized counts
hPTMs enrichment
ChIP-seq
Correlation:
All P < 0.001
H3K9ac:
rho = 0.41
H3K27ac:
rho = 0.43
H3K4me3:
rho = -0.11
H3K9me3
rho = -0.28
Linear regression:
R2 ~ 27%
H3K9ac: 9%
H3K27ac:13%
H3K4me3: 0%
H3K9me3: 5%
RNA-seq Ctrl.
(Log2)
10
0
-10
ChIP-seq
Ctrl. (Log2)
4.1
0
-3.2
H3K9ac
H3K27ac
H3K4me3
H3K9me3
B
hPTMs Control
log2 FC ChIP/Input
H3K9ac
H3K27ac
H3K4me3
H3K9me3
1.0
0.0
-1.0
Colorkey
−3
−1
0
1
2
H3K9ac
H3K27ac
H3K4me3
H3K9me3
Intron %
Exon %
−2Kb 5' 33% 66% 3' +2Kb
Intron relative length
High
(N=3448)
0.5
-0.5
Medium
(N=3197)
1.0
-1.0
Low
(N=2996)
1.0
-1.0
-2Kb 5' 3' +2Kb
C
Infection
Control
8234
30832
14138

Figure S2

A

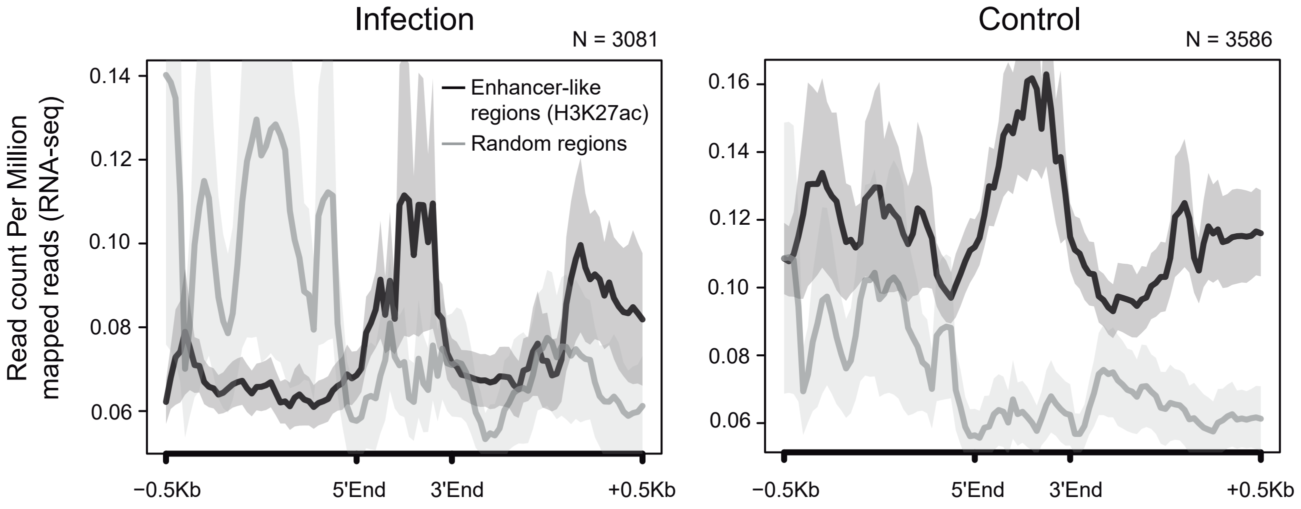

Infection
N = 3081
Control
N = 3586
Read count Per Million mapped reads (RNA-seq)
Enhancer-like regions (H3K27ac)
Random regions
0.14
0.12
0.10
0.08
0.06
0.16
−0.5Kb
5'End
3'End
+0.5Kb

Figure S3

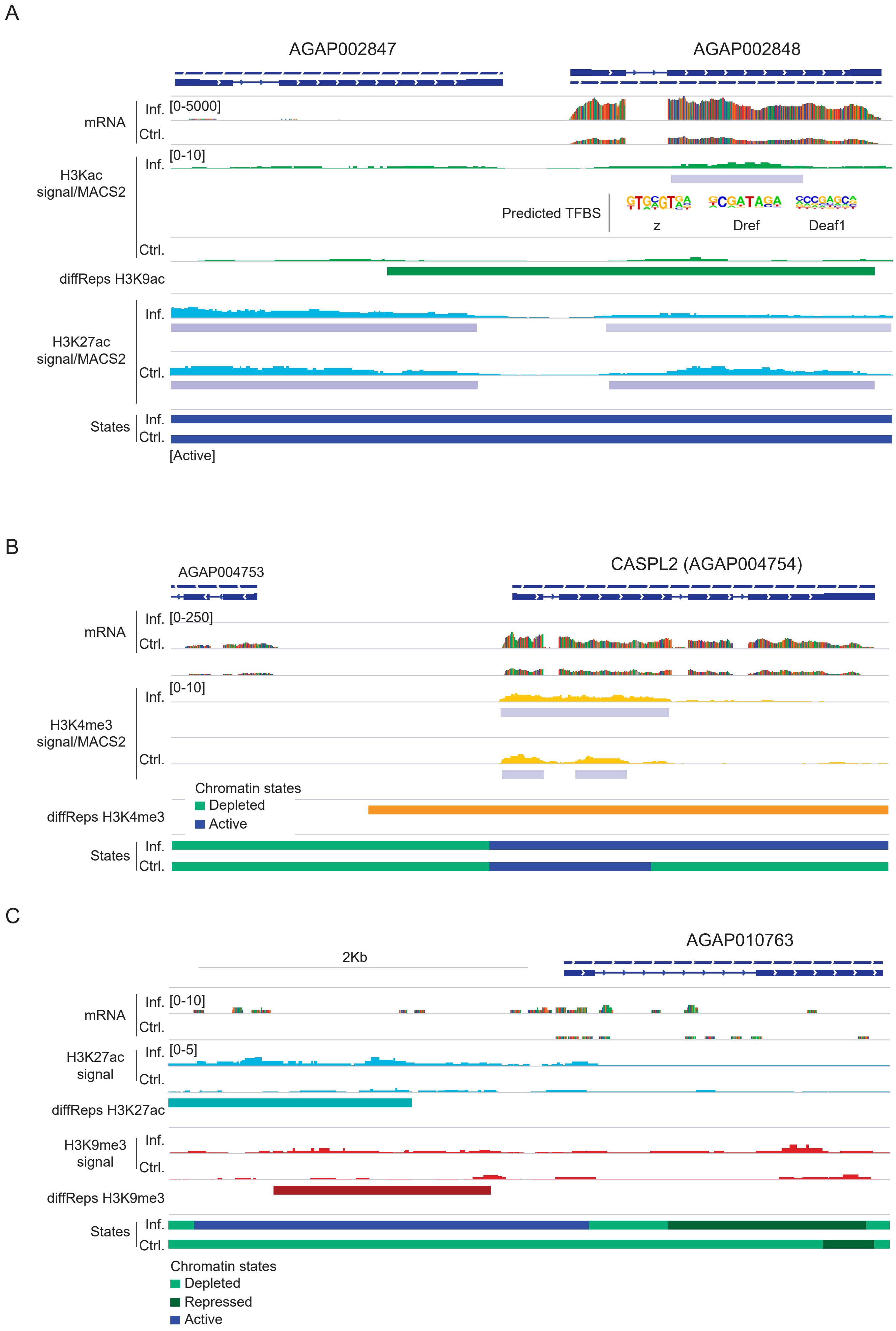
A
AGAP002847
AGAP002848
mRNA
Inf.
[0-5000]
Ctrl.
H3Kac
signal/MACS2
Inf.
[0-10]
Predicted TFBS
z
Dref
Deaf1
Ctrl.
diffReps H3K9ac
H3K27ac
signal/MACS2
Inf.
Ctrl.
States
Inf.
Ctrl.
[Active]
B
AGAP004753
CASPL2 (AGAP004754)
mRNA
Inf.
[0-250]
Ctrl.
H3K4me3
signal/MACS2
Inf.
[0-10]
Ctrl.
Chromatin states
Depleted
Active
diffReps H3K4me3
States
Inf.
Ctrl.
C
AGAP010763
2Kb
mRNA
Inf.
[0-10]
Ctrl.
H3K27ac
signal
Inf.
[0-5]
Ctrl.
diffReps H3K27ac
H3K9me3
signal
Inf.
Ctrl.
diffReps H3K9me3
States
Inf.
Ctrl.
Chromatin states
Depleted
Repressed
Active

Figure S4

A

Mfuzz analysis on histone modification enrichment ratios in diffReps-annotated genes

Clusters definition:

* Max. value (signal peak Infection)

▼ Min. value (signal peak Control)

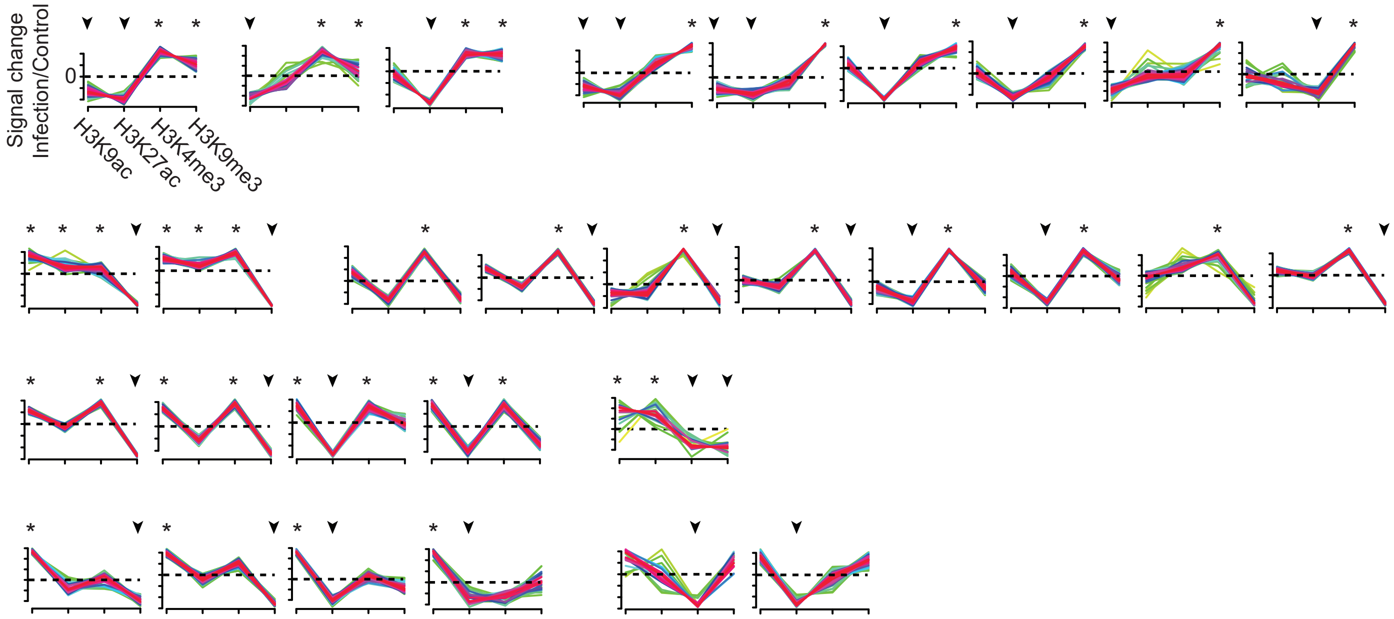

Clusters Infection - patterns: (*)

H3K9ac
H3K9ac/K27ac
H3K9ac/K4me3
H3K4me3
H3K9ac/K27ac/K4me3
H3K9me3
H3K9me3/K4me3

Clusters Control - patterns: (▼)

H3K9ac
H3K9ac/K27ac
H3K27ac
H3K4me3
H3K9me3
H3K9me3/K4me3

A

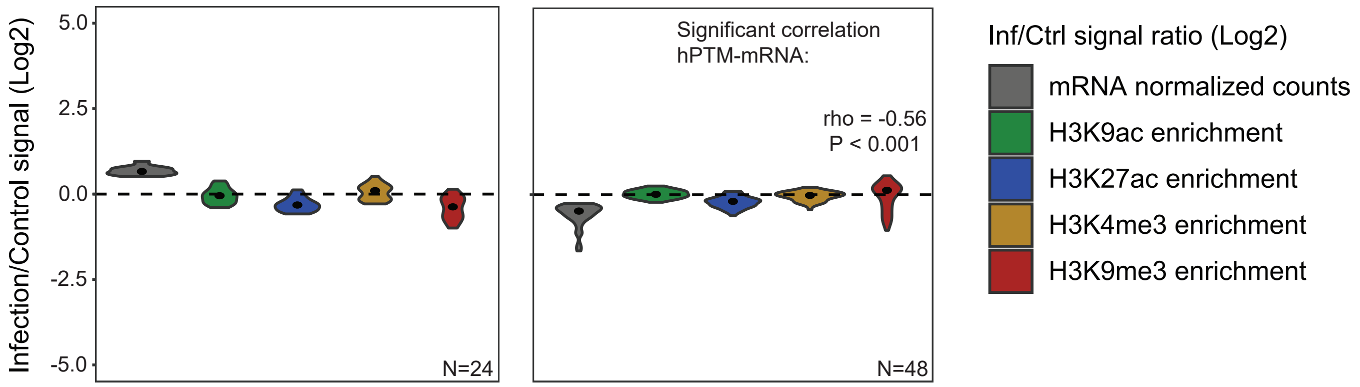

Infection/Control signal (Log2)
5.0
2.5
0.0
-2.5
-5.0
N=24
Significant correlation
hPTM-mRNA:
rho = -0.56
P < 0.001
N=48
Inf/Ctrl signal ratio (Log2)
mRNA normalized counts
H3K9ac enrichment
H3K27ac enrichment
H3K4me3 enrichment
H3K9me3 enrichment

Figure S6

A

Mfuzz analysis on histone modification enrichment ratios in significant differentially expressed genes (DESeq2)

Clusters definition: (Infection)

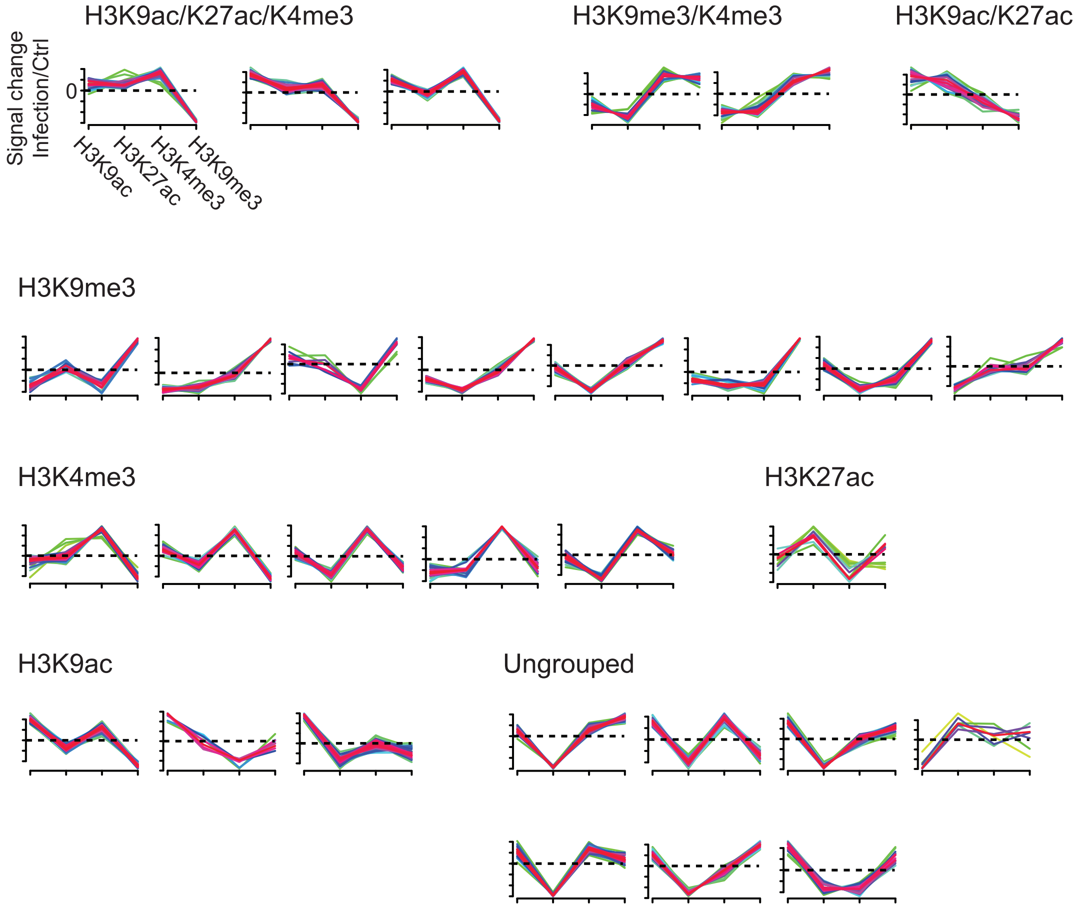

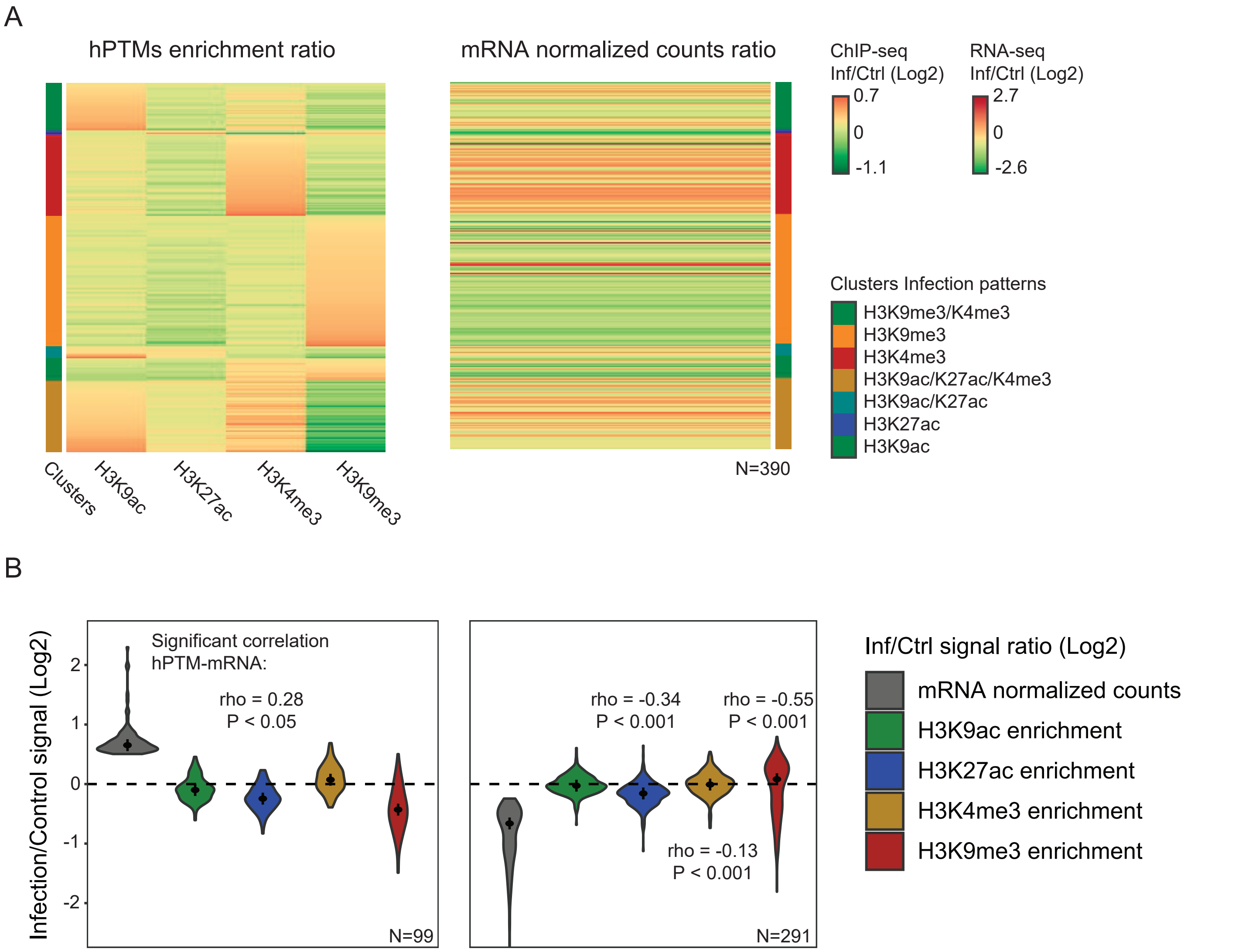
A
hPTMs enrichment ratio
mRNA normalized counts ratio
ChIP-seq
Inf/Ctrl (Log2)
0.7
0
-1.1
RNA-seq
Inf/Ctrl (Log2)
2.7
0
-2.6
Clusters Infection patterns
H3K9me3/K4me3
H3K9me3
H3K4me3
H3K9ac/K27ac/K4me3
H3K9ac/K27ac
H3K27ac
H3K9ac
Clusters
H3K9ac
H3K27ac
H3K4me3
H3K9me3
N=390
B
Infection/Control signal (Log2)
Significant correlation
hPTM-mRNA:
rho = 0.28
P < 0.05
N=99
rho = -0.34
P < 0.001
rho = -0.55
P < 0.001
rho = -0.13
P < 0.001
N=291
Inf/Ctrl signal ratio (Log2)
mRNA normalized counts
H3K9ac enrichment
H3K27ac enrichment
H3K4me3 enrichment
H3K9me3 enrichment

Figure S8

SUPPLEMENTAL FIGURES LEGENDS

FIGURE S1

A. Spearman correlation plots between histone modifications and input ChIP-seq libraries. Libraries are subsampled to equal number of reads (9 M) and ordered following a hierarchical clustering approach.

B. Annotation of MACS2 ChIP-seq peaks for each histone modification to genomic features: TSS-Promoters, TTSs, Intergenic, Intron and Exon regions. The plot corresponds to control condition.

C. Density plot showing the position (Kb upstream) of MACS2 peaks for each histone modification and control condition with respect to the ATG initiation codon of the nearest downstream gene.

D. Heatmap of emission parameters from ChromHMM analysis using a four chromatin states model based on histone modification enrichment patterns in the control condition. The predicted states are: Deplet (depleted, low levels of all hPTMs), Repr (repressive, H3K9me3 enrichment), Biv (bivalent, H3K4me3/H3K9me3 enrichment) and Act (active, H3K27ac/H3K9ac/H3K4me3 enrichment). Darker blue indicates higher enrichment of a particular histone modification.

E. Heatmap showing the overlap of various genomic features, including MACS2 peaks located in promoters (-2 Kb from the ATG) or gene bodies in the control condition, with the predicted chromatin states. Darker blue in the first column indicates higher percentage of the genome overlapped by a given state. For other columns, it indicates the likelihood of finding a particular chromatin state in each genomic feature compared to what it would be expected by chance.

FIGURE S2

A. Heatmaps showing mRNA levels (left) and histone modification enrichment profiles (right), of genes displaying a MACS2 peak in the promoter or the gene body. Data correspond to the control condition. Genes are ordered by mRNA levels. ChIP-seq enrichment at the promoters and gene bodies is normalized (RPKM) and input-corrected. Data are log2-scaled and mean-centered.

Spearman rank correlation coefficient (rho) and corresponding P value are shown for the association between each histone modification enrichment levels and mRNA levels. The variance in mRNA levels explained by combined and individual histone modifications is shown, according to a linear regression model considering gene expression as response and ChIP-seq enrichment as covariates.

B. Heatmaps showing histone modification enrichment profiles at high and medium expressed genes in the control condition. Genes are ordered by the percentage of the bodies containing introns and exons. Average profile plots show density of normalized (RPKM) and input corrected ChIP-seq reads for each histone modification at high and medium expressed genes (top) and at those genes classified by the percentage of gene bodies containing introns (right).

C. Venn diagram showing the overlap between MACS2 peaks identified in infected and control conditions. The area is proportional to the number of peaks.

FIGURE S3

Profile plots showing RNA-seq signal at the distal regions displaying significant enrichment in H3K27ac (MACS2 peaks), both at infected (left) and control (right) condition. The graphs represent Read counts Per Million (RPM) mapped RNA-seq reads in a window surrounding the regions. Profiles in gray represent signal at random coordinates.

FIGURE S4

A-C. Histone modification enrichment profiles in regions containing Niemann-Pick Type C-2 genes (AGAP002847-AGAP002848), CASPL2 (AGAP004754) and fibrinogen-related protein (AGAP010763) encoding genes. Tracks show normalized/input-corrected ChIP-seq signals and RNA-seq mapped read counts for each condition. The location of diffReps regions, MACS2 peaks, predicted transcription factor binding sites and predicted chromatin states for each condition are included. All tracks are shown at equal scale.

FIGURE S5

A. Histone modification enrichment profiles for clusters identified in the Mfuzz soft clustering analysis. Signal is computed as the ratio of normalized (RPKM) input-corrected ChIP-seq reads in infected relative to the control condition at promoters and genes bodies containing high-confidence diffReps regions. Data are log2-scaled. Yellow/green colored profiles represent regions with low membership and red/purple elements with high membership value to each cluster. Clusters of regions for infected and control conditions are defined based on the Mfuzz groups that share unique histone modification enrichment patterns. An asterisk (*) marks histone modifications showing peaks of enrichment in the infected condition. Histone modifications showing peaks of enrichment in the control condition are marked with arrows (➤).

FIGURE S6

A. Ratio of gene expression and histone modification enrichments between infected and control conditions for genes with significant differential gene expression (DESeq2) and containing high-confidence diffReps regions in the promoter or the gene body. Genes are divided by more highly expressed in infected (left) and uninfected (right) mosquito tissues. ChIP-seq enrichment at promoters and gene bodies is normalized (RPKM) and input-corrected. Data are the log2-scaled ratio of mRNA and histone modification enrichment levels between the infected and the control conditions. Spearman rank correlation coefficient (rho) and corresponding P value are shown for significant correlations between histone modification enrichments and mRNA levels.

FIGURE S7

A. Histone modification enrichment profiles for clusters identified in the Mfuzz soft clustering analysis. Signal is computed as the ratio of normalized (RPKM) input-corrected ChIP-seq reads in

the infected relative to the control condition for genes showing significant differential levels of expression according to DESeq2 analysis. Data are log2-scaled. Yellow/green colored profiles represent regions with low membership and red/purple elements with high membership value to each cluster. Clusters are classified in groups that share unique histone modification enrichment patterns. Some clusters showing unspecific profiles remain ungrouped.

FIGURE S8

A. Heatmaps showing clusters of genes (-2 Kb) grouped by unique histone modification profiles identified in the soft clustering analysis (left) on DESeq2 genes and corresponding changes in mRNA levels (right). ChIP-seq enrichment at promoters and gene bodies is normalized (RPKM) and input-corrected. The signal corresponds to the ratio of ChIP-seq and mRNA levels in the infected versus the control condition. Data are log2-scaled and mean-centered.

B. Ratio of gene expression and histone modification enrichment between infected and control conditions for DESeq2 gene clusters more highly expressed in infected (left) and control (right) conditions. Data are the log2-scaled ratio between the infected and the control as in (A). Spearman rank correlation coefficient (rho) and corresponding P value are shown for significant correlations between histone modification enrichments and mRNA levels.
